# Quantum spin liquid in the semiclassical regime

**DOI:** 10.1038/s41467-018-03934-1

**Published:** 2018-04-23

**Authors:** Ioannis Rousochatzakis, Yuriy Sizyuk, Natalia B. Perkins

**Affiliations:** School of Physics and Astronomy, University of Minnesota, Minneapolis, MN 55455 USA

## Abstract

Quantum spin liquids (QSLs) have been at the forefront of correlated electron research ever since their proposal in 1973, and the realization that they belong to the broader class of intrinsic topological orders. According to received wisdom, QSLs can arise in frustrated magnets with low spin *S*, where strong quantum fluctuations act to destabilize conventional, magnetically ordered states. Here, we present a *Z*_2_ QSL ground state that appears already in the semiclassical, large-*S* limit. This state has both topological and symmetry-related ground-state degeneracy, and two types of gaps, a “magnetic flux” gap that scales linearly with *S* and an “electric charge” gap that drops exponentially in *S*. The magnet is the spin-*S* version of the spin-1/2 Kitaev honeycomb model, which has been the subject of intense studies in correlated electron systems with strong spin–orbit coupling, and in optical lattice realizations with ultracold atoms.

## Abstract (web summary)

Strongly correlated quantum spin liquid phases form when quantum fluctuations prevent magnetic ordering, which normally requires low spin systems that cannot be analyzed semiclassically. Here, the authors show that a large-spin Kitaev model supports a spin liquid phase in the semiclassical limit.

## Introduction

Quantum spin liquids (QSLs) describe systems that evade magnetic long-range order down to zero temperature, and manifest a number of remarkable phenomena, such as topological ground-state degeneracies, emergent gauge fields, and fractional excitations with non-trivial statistics^1–11^. The rich phenomenology of QSLs derives from an intrinsic tendency to form massive quantum superpositions of local, product-like wavefunctions. Notable examples are the resonating valence bond (RVB) state^1, 5, 12–14^, the gapped QSL of the Toric code^6^, and the gapless QSL phase of the spin-1/2 Kitaev honeycomb model^7^. Typically, such massive superpositions arise in frustrated magnets with low spin *S*, which ideally have an infinite number of competing states and a strong tunneling between them^15^.

Here, we show that the spin-*S* version of the celebrated Kitaev honeycomb model^7^ is a topological *Z*_2_ QSL already in the semiclassical limit. Specifically, the leading semiclassical fluctuations give rise to an effective low-energy description in terms of a pseudospin-1/2 Toric code^6^. The “magnetic flux” term of the Toric code arises from the zero-point energy of spin waves above the classical ground states, while the “electric charge” term stems from the tunneling between different classical states. The ensuing *Z*_2_ QSL lives on top of a honeycomb superlattice of “frozen” spin dimers^16^, which take only two possible configurations, instead of (2*S* + 1)^2^. These two states are the pseudospin-1/2 degrees of freedom of the Toric code. The frozen dimer pattern breaks translational symmetry, so the QSL possesses an extra degeneracy associated to symmetry breaking, besides the topological one. We also show that the large-*S* description breaks down around *S* ~ 3/2. For lower *S*, tunneling processes that shift the dimer positions become quickly relevant and compete with the “freezing” energy scale *δE*_f_. Including these processes lead to a picture of “decorated quantum dimers”, where both the dimer positions and the orientations of the two spins in each dimer are allowed to resonate. The resulting picture for *S* = 1 in terms of another type of spin liquid will be discussed.

## Results

### Model and classical ground states

The spin-*S* Kitaev model on the honeycomb lattice is described by the Hamiltonian

1 $${\cal H} = K\left( {\mathop {\sum}\limits_{\langle ij\rangle \in \,\textrm{\textquotedblleft x \textquotedblright }} S_i^xS_j^x + \mathop {\sum}\limits_{\langle ij\rangle \in \,\textrm{\textquotedblleft y \textquotedblright}} {\kern 1pt} S_i^yS_j^y + \mathop {\sum}\limits_{\langle ij\rangle \in \,\textrm{\textquotedblleft z \textquotedblright}} {\kern 1pt} S_i^zS_j^z} \right).$$

**Fig. 1:**
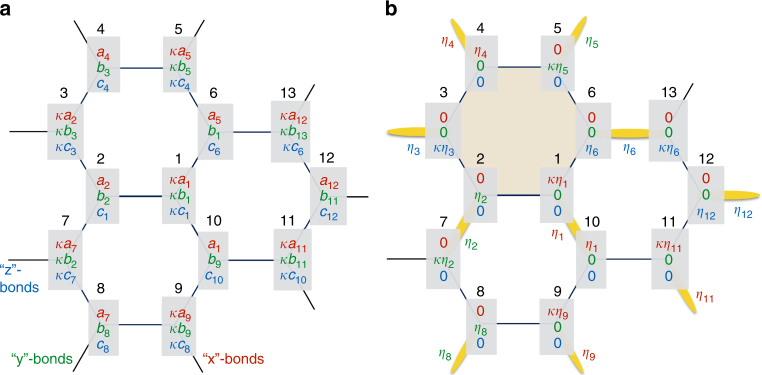
Classical ground states of the Kitaev model on the honeycomb lattice. The lattice has three types of NN bonds, labeled by “**x**”, “**y**”, and “**z**”. **a** General form of ground states. The numbers 1–13 label the spin sites, and the shaded 3×1 vectors below each number give the corresponding Cartesian components (*x*, *y*, and *z*) of the spins (colored red, green, and blue, respectively). Here, *κ* = −sgn(*K*), **S**_*i*_ = (*a*_*i*_, *b*_*i*_, *c*_*i*_) or *κ*(*a*_*i*_, *b*_*i*_, *c*_*i*_) if *i* belongs to the A or B sublattice, and $a_i^2 + b_i^2 + c_i^2 = S^2$. **b** The so-called Cartesian states of BSS correspond to the states where only one of the Cartesian component is finite. These states map to dimer coverings, with (yellow) dimers representing satisfied bonds. The spin orientation of each dimer is described by an Ising-like variable *η* = ±1 (colored according to the non-vanishing Cartesian component of the two spins shared by the bond). The shaded hexagon has the shortest loop with no dimers

Here, $\left\langle {ij} \right\rangle$ denotes a pair of nearest-neighbor (NN) spins **S**_*i*_ and **S**_*j*_. There are three types of NN bonds, depending on their orientation, which are labeled by “**x**”, “**y**” or “**z**” (Fig. 1a). These bonds are directed along $\frac{{\mathbf{y - z}}}{{\sqrt 2 }}$, $\frac{{\mathbf{z - x}}}{{\sqrt 2 }}$, and $\frac{{\mathbf{x - y}}}{{\sqrt 2 }}$, respectively, where **x**, **y**, and **z** are the usual unit vectors in the Cartesian frame. The constant *K* denotes the Kitaev interaction. Note that there is a four sublattice duality transformation^17^ that maps the positive *K* to the negative *K* model, but we shall discuss the general case here for completeness. We shall also define *κ* = −sgn(*K*).

The classical ground states of this model were first analyzed by BSS^16^. There, the authors identified an infinite number of so-called “Cartesian” states, which map to dimer coverings of the honeycomb lattice, modulo a factor of two for the orientation of the two spins per dimer. They further showed that the Cartesian states are connected to each other by continuous valleys of other ground states, leading to a huge ground-state degeneracy. Soon after, Chandra et al.^18^ showed that the manifold actually consists of infinitely more ground states and possesses an emergent gauge structure that leads to power law correlations.

The crucial aspect of the present study is the use of a convenient parametrization of the classical ground-state manifold, which reveals the topological terms arising from quantum fluctuations in an explicit way. This parametrization is shown in Fig. 1a. We denote the two sublattices of the honeycomb by A and B. Next, we parametrize each spin as **S**_*i*_ = (*a*_*i*_, *b*_*i*_, *c*_*i*_) or *κ*(*a*_*i*_, *b*_*i*_, *c*_*i*_) for *i* ∈ A or B, respectively, and $a_i^2 + b_i^2 + c_i^2 = S^2$. Then, for every pair of NN sites, **S**_*i*_ and **S**_*j*_, we can minimize their mutual interaction by requiring that *a*_*i*_ = *a*_*j*_ or *b*_*i*_ = *b*_*j*_ or *c*_*i*_ = *c*_*j*_, if the two sites share, respectively, an “**x**” or “**y**“ or “**z**” type of bond. To see if the ensuing states are ground states, we check that they saturate the lower bound of the energy per site, $E_{{\mathrm{min}}}{\mathrm{/}}N = - \left| K \right|S^2{\mathrm{/}}2$^16^. Indeed, the energy from the three bonds emanating from any site *i* add up to $- \left| K \right|\left( {a_i^2 + b_i^2 + c_i^2} \right) = - \left| K \right|S^2$. And since each bond is shared by two sites, these configurations saturate the lower bound and are therefore ground states. The Cartesian states of BSS arise by keeping only one component of (*a*_*i*_, *b*_*i*_, *c*_*i*_) finite, and equal to *η*_*i*_*S*, where *η*_*i*_ = ±1. Modulo these Ising-like variables, the Cartesian states map to dimer coverings of the lattice [Fig. 1b]. There are (1.381)^*N*/2^ coverings^19–21^, and (1.662)^*N*^ Cartesian states in total^16^.

The semiclassical analysis leading to the Toric code proceeds in three steps. The first is to show that fluctuations select the Cartesian over the non-Cartesian states, which identifies the positions and spin orientations of the dimers as the relevant degrees of freedom. In the second step, which was carried out by BSS^16^, fluctuations freeze the positions of the dimers to a given pattern, leaving their spin orientation as the only relevant degrees of freedom below the associated freezing energy scale *δE*_f_. These degrees of freedom can be described by pseudospin-1/2 variables residing on the bonds of a honeycomb superlattice. This parametrization reveals that the order by disorder effect has a topological structure that was not noticed previously, and which is robust to all orders in 1/*S* expansion. The third step is to go beyond the order by disorder effect and include quantum mechanical tunneling between different pseudospin configurations. This step is essential for restoring all local *Z*_2_ gauge symmetries of the original model, and for the formation of the quantum spin liquid.

### Selection of Cartesian states

The first crucial ingredient of the effective description in terms of dimers is to show that fluctuations select the Cartesian over the non-Cartesian states. BSS made this hypothesis based on an analogy to a related 1D problem. Here, we prove it by real space perturbation theory (RSPT)^22–25^. We introduce local frames (***u***_*i*_, **v**_*i*_, **w**_*i*_), with **w**_*i*_ along the classical directions, and write ${\bf{S}}_i = S_i^w{\bf{w}}_i + S_i^u{\bf{u}}_i + S_i^v{\bf{v}}_i$. Then, we split ${\cal H}$ into a diagonal part ${\cal H}_0 = h\mathop {\sum}\nolimits_i \left( {S - S_i^w} \right)$, describing fluctuations in the local field *h* = *KS*, and a perturbation ${\cal V} = {\cal H} - {\cal H}_0$, which couples fluctuations on different sites. The essential physics is captured by the leading, short-wavelength corrections from second-order RSPT. The three types of bonds, say (1–10), (1–6), and (1–2) of Fig. 1, give $\delta E_{1,10} = \xi \left( {1 - \tilde a_1^2} \right)^2$, $\delta E_{1,6} = \xi \left( {1 - \tilde b_1^2} \right)^2$, $\delta E_{1,2} = \xi \left( {1 - \tilde c_1^2} \right)^2$, where $\xi = - \left| K \right|S{\mathrm{/}}8$ and $\left( {\tilde a_i,\tilde b_i,\tilde c_i} \right) = \left( {a_i,b_i,c_i} \right){\mathrm{/}}S$. Using the spin length constraints and disregarding overall constants, gives the anisotropy term

2 $$\delta E_{{\mathrm{ani}}} = - \left( {\left| K \right|S{\mathrm{/}}16} \right)\mathop {\sum}\nolimits_i \left( {\tilde a_i^4 + \tilde b_i^4 + \tilde c_i^4} \right),$$

similar to the one found in refs. ^26, 27^. This anisotropy selects the Cartesian states, confirming the hypothesis of BSS^16^.

### Dimer freezing and ***η***-variables

**Fig. 2:**
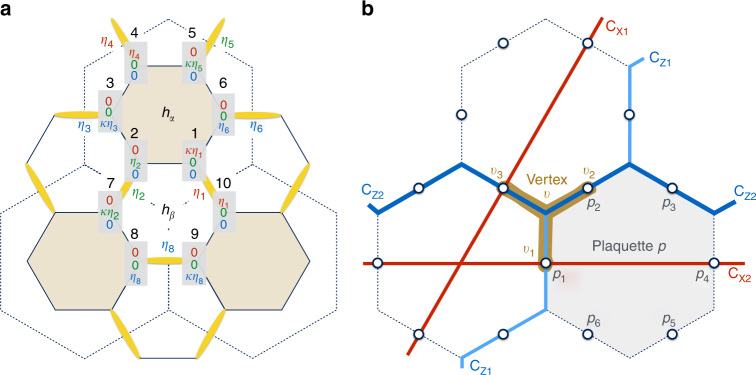
The mapping to a Toric code on a honeycomb superlattice. **a** Star dimer pattern selected from spin waves^16^. The numbers 1–10 label the spin sites, and the shaded 3×1 vectors below each number give the corresponding Cartesian components. The star pattern is one of the Cartesian states of Fig. 1b, which map to dimer (yellow) coverings on the lattice. The *η*’s describe the orientation of the two spins per dimer. They sit at the middle of the bonds of a honeycomb superlattice (dashed). **b** The resulting Toric code description of Eq. (10) on the honeycomb superlattice. The three- and six-body operators **A**_*v*_ and **B**_*p*_ of Eq. (10) are defined on vertices *v* and plaquettes *p* of the superlattice. These connect, respectively, three sites (*v*_1_–*v*_3_) and six sites (*p*_1_–*p*_6_) of the superlattice. For the torus geometry, C_X1_ and C_X2_ (similarly for C_Z1_ and C_Z2_) are non-contractible loops that wrap the system in different directions

Next, we discuss the lifting of the degeneracy within the manifold of Cartesian states, starting with the corrections from spin waves. As shown by BSS, the linear spin-wave Hamiltonian splits into non-interacting modes propagating along loops without dimers, and the minimum zero-point energy arises by maximizing the number of the shortest such “empty” loops, like the shaded hexagon of Fig. 1b. This gives the “star” or “columnar” dimer pattern of Fig. 2a, which is known from the context of the quantum dimer model and the frustrated Heisenberg model on the honeycomb lattice^28–30^. In this pattern, the only dynamical degrees of freedom remaining are the Ising-like variables *η* = ±1, which specify the direction of the two spins shared by each given dimer.

The physics of the dimer freezing is actually more involved from what is predicted from the linear spin-wave theory, but let us postpone this discussion for later and focus on the spin states associated to the “star” pattern. There are three ways to place this pattern in the lattice and each dimer has two configurations, so at first sight, the number of selected spin states is 3 × 2^*N*/2^. BSS showed, however, that the minimum zero-point energy is associated with spin-wave modes that have antiperiodic boundary conditions (ABC) around the empty hexagons, which reduces the number of states to 3 × 2^*N*/3^.

However, this is not the full story yet. It turns out that the boundary condition on the spin-wave modes actually endows the selected manifold with a topological magnetic flux term (and, in particular, the above number of states has to be multiplied 2^2*g*−1^, where *g* is the genus of the system). To see this, we repeat the spin-wave analysis using our *η*-parametrization. We begin by rewriting ${\cal H}$ in the local frame. Let us take the empty hexagon *h*_*α*_ of Fig. 2a and choose *u*_*i*_ and *v*_*i*_ in the following way (and similarly for every other empty hexagon):

3 $$\begin{array}{*{20}{l}} {{\bf{w}}_1 = \kappa \eta _1{\bf{x}},} \hfill & {{\bf{u}}_1 = - \kappa \eta _1{\bf{z}},} \hfill & {{\bf{v}}_1 = {\bf{y}},} \hfill \\ {{\bf{w}}_2 = \eta _2{\bf{y}},} \hfill & {{\bf{u}}_2 = - \kappa \eta _1{\bf{z}},} \hfill & {{\bf{v}}_2 = - \kappa \eta _1\eta _2{\bf{x}},} \hfill \\ {{\bf{w}}_3 = \kappa \eta _3{\bf{z}},} \hfill & {{\bf{u}}_3 = \eta _1\eta _2\eta _3{\bf{y}},} \hfill & {{\bf{v}}_3 = - \kappa \eta _1\eta _2{\bf{x}},} \hfill \\ {{\bf{w}}_4 = \eta _4{\bf{x}},} \hfill & {{\bf{u}}_4 = \eta _1\eta _2\eta _3{\bf{y}},} \hfill & {{\bf{v}}_4 = \eta _1\eta _2\eta _3\eta _4{\bf{z}},} \hfill \\ {{\bf{w}}_5 = \kappa \eta _5{\bf{y}},} \hfill & {{\bf{u}}_5 = - \kappa \eta _1\eta _2\eta _3\eta _4\eta _5{\bf{x}},} \hfill & {{\bf{v}}_5 = \eta _1\eta _2\eta _3\eta _4{\bf{z}},} \hfill \\ {{\bf{w}}_6 = \eta _6{\bf{z}},} \hfill & {{\bf{u}}_6 = - \kappa \eta _1\eta _2\eta _3\eta _4\eta _5{\bf{x}},} \hfill & {{\bf{v}}_6 = - \kappa B_{h_\alpha }{\bf{y}},} \hfill \end{array}$$

where **x**, **y**, and **z** are the Cartesian unit vectors, and the product of the six ***η***-variables on empty hexagons,

4 $$B_{h_\alpha } = \eta _1\eta _2\eta _3\eta _4\eta _5\eta _6,$$

is the magnetic flux that plays a central role in the following. With the above choice of the local frames, the couplings between empty hexagons map to terms of the type $\kappa S_i^wS_j^w$. For example, $S_1^xS_{10}^x \mapsto \kappa S_1^wS_{10}^w$. On the other hand, the intra-hexagon terms map as follows

5 $$\begin{array}{*{20}{l}} {S_1^zS_2^z \mapsto S_1^uS_2^u, } \hfill & {S_2^xS_3^x \mapsto S_2^vS_3^v,} \hfill \\ {S_3^yS_4^y \mapsto S_3^uS_4^u, } \hfill & {S_4^zS_5^z \mapsto S_4^vS_5^v,} \hfill \\ {S_5^xS_6^x \mapsto S_5^uS_6^u, } \hfill & {S_6^yS_1^y \mapsto - \kappa B_{h_\alpha }S_6^vS_1^v.} \hfill \end{array}$$

Thus, in the rotated frame, the only dependence of the Hamiltonian on *η*’s is via the fluxes $\left\{ {B_{h_\alpha }} \right\}$ on the empty hexagons {*h*_*α*_}. And, since the choice of the local frame does not alter the physics, it follows that classical states that belong to the “star” pattern of Fig. 2a and have the same $\left\{ {B_{h_\alpha }} \right\}$ share the same semiclassical spin-wave spectrum, at all orders in 1/*S*. The same is true for the renormalization of the ground-state energy and therefore the order by-disorder effect.

Let us show the latter explicitly and we shall return to the spin-wave modes further below. We introduce the usual Holstein–Primakoff bosons *c*_*i*_ via the transformation^31^, $S_i^\dagger = S_i^u + iS_i^v = \left( {2S - c_i^\dagger c_i} \right)^{1/2}c_i$, $S_i^w = S - n_i$, $n_i = c_i^\dagger c_i$. The coupling between neighboring empty hexagons, like $S_1^xS_{10}^x$, reduces then to

6 $$S_1^xS_{10}^x \mapsto \kappa S_1^wS_{10}^w = \kappa \left( {S - n_1} \right)\left( {S - n_{10}} \right).$$

**Fig. 3:**
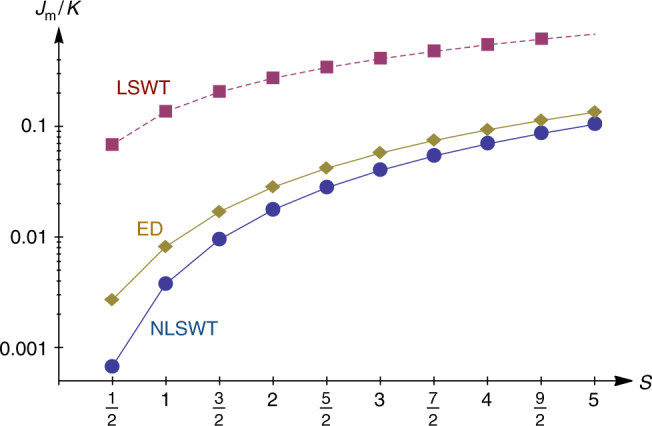
Evolution of *J*_m_ as a function of *S*. The three curves are extracted from linear spin-wave theory (LSWT, red squares), non-linear spin-wave theory (NLSWT, blue circles), and exact diagonalizations (ED, dark yellow diamonds) of the Hamiltonian ${\cal H}_{{\mathrm{MF}}}$ discussed in the “Methods” section

The linear spin-wave theory amounts to disregarding the term *κn*_1_*n*_10_ from the right-hand side of this equation. Empty hexagons then decouple, leading to a quadratic, six-site boson problem, with two sublattices and periodic (PBC) or antiperiodic (ABC) boundary conditions, for $\kappa B_{h_\alpha } = - 1$ or 1, respectively. So, the BSS result that ABC give the lowest zero-point energy amounts to imposing $\kappa B_{h_\alpha } = 1$ for all empty hexagons *h*_*α*_. More explicitly, by combining the zero-point energies, *δE*_PBC_ and *δE*_ABC_ for PBC and ABC, respectively, we get, for a given *h*_*α*_,

7 $$\delta E\left( {h_\alpha } \right) = c + J_{\mathrm{m}}\eta _1\eta _2\eta _3\eta _4\eta _5\eta _6 = c + J_{\mathrm{m}}B_{h_\alpha },$$

where $c = \frac{{\delta E_{{\mathrm{PBC}}} + \delta E_{{\mathrm{ABC}}}}}{2}$ and $J_{\mathrm{m}} = \frac{{\delta E_{{\mathrm{PBC}}} - \delta E_{{\mathrm{ABC}}}}}{2}$. The linear spin-wave theory of BSS^16^ gives $\delta E_{{\mathrm{PBC}}} = 2\left| K \right|S$ and $\delta E_{{\mathrm{ABC}}} = \sqrt 3 \left| K \right|S$, and so $J_{\mathrm{m}} = \frac{{2 - \sqrt 3 }}{2}KS$. However, as shown in Fig. 3 and emphasized below, the linear theory overestimates $\left| {J_{\mathrm{m}}} \right|$ strongly due to the presence of a large percentage (four out of six) of “spurious” zero modes.

Equation (7) confirms that the ground-state energy depends explicitly on the fluxes $\left\{ {B_{h_\alpha }} \right\}$, and that states with the same set of fluxes have the same zero-point energy. Importantly, the quartic terms of the type *n*_1_*n*_10_ of Eq. (6) will give rise to interactions between the fluxes, of the form $J_{\alpha \alpha {\prime}}B_{h_\alpha }B_{h_{\alpha {\prime}}}$ plus higher-order terms, where the couplings *J*_*αα*′_ depend on the positions of the corresponding empty hexagons *h*_*α*_, *h*_*α*′_. As we demonstrate further below, these interactions are at least one order of magnitude weaker than the leading term ∝*J*_m_, and in addition do not alter any of the crucial ingredients leading to a topological QSL state.

With this in mind, we are now ready to identify the first crucial ingredient of the Toric code description announced above. The *η*_*i*_ variables live on the midpoints of the bonds of a honeycomb superlattice (Fig. 2a) and we can promote these variables to Pauli matrices ${\boldsymbol{\eta }}_i^z$. Then the dominant, non-trivial correction to the zero-point energy—the second term of the right-hand side of Eq. (7)—is the magnetic flux term of the Toric code^6^ on this superlattice.

### Quantum mechanical tunneling

The second ingredient of the Toric code, the electric charge term, stems from processes that flip the three *η*’s around a vertex of the superlattice. Let us take, e.g., the spin coherent state of the *h*_*β*_ hexagon of Fig. 2a,

8 $$\left| {h_\beta } \right\rangle = \left| {\kappa \eta _8{\bf{z}}} \right\rangle _9\left| {\eta _8{\bf{z}}} \right\rangle _8\left| {\kappa \eta _2{\bf{y}}} \right\rangle _7\left| {\eta _2{\bf{y}}} \right\rangle _2\left| {\kappa \eta _1{\bf{x}}} \right\rangle _1\left| {\eta _1{\bf{x}}} \right\rangle _{10}.$$

The leading processes that transform this state to its time-reversed state $\left| {\bar h_\beta } \right\rangle$, with *η*_1_, *η*_2_, and *η*_8_ flipped, appear in (6*S*)-th order of RSPT, with ${\cal V} = K\left( {S_7^xS_8^x + S_9^yS_{10}^y + S_1^zS_2^z} \right)$. The corresponding off-diagonal matrix element *J*_e_ of the resulting effective Hamiltonian ${\cal H}_{{\mathrm{eff}}}$ depends, unlike *J*_m_, on the choice of the local axes (**u**_*i*_, **v**_*i*_). Here, we fix *J*_e_ to be a real number by choosing the local axes such that ${\cal V} \mapsto K\left( {S_7^uS_8^u + S_9^uS_{10}^u + S_1^uS_2^u} \right)$. Following, e.g., the steps of the Supplementary Material of ref. ^26^, we get

9 $$\left\langle {\bar h_\beta \left| {{\cal H}_{{\mathrm{eff}}}} \right|h_\beta } \right\rangle \equiv J_{\mathrm{e}} = 3 \times 2^{5 - 18S}S^{5 - 6S}\left[ {(2S - 1)!} \right]^3K.$$

In the language of the ***η*** operators, this matrix element is represented by $J_{\mathrm{e}}{\boldsymbol{\eta }}_1^x{\boldsymbol{\eta }}_2^x{\boldsymbol{\eta }}_8^x \equiv J_{\mathrm{e}}{\bf{A}}_v$, which involve the three *η*’s around the vertex *v* that sits at the center of *h*_*β*_ (Fig. 2a).

Similarly to the higher-order potential terms mentioned above, there also exist higher-order tunneling terms, such as **A**_*v*_**A**_*v*′_, whose amplitudes are much weaker than *J*_e_.

### Effective Hamiltonian of ***η***-variables

Collecting the potential energy (disregarding *c*) and the tunneling terms above gives the effective Hamiltonian for the ***η***-variables:

10 $$\begin{array}{*{20}{l}} {{\cal H}_{{\mathrm{eff}}}} \hfill & = \hfill & {J_{\mathrm{e}}\mathop {\sum}\nolimits_v {\kern 1pt} {\boldsymbol{\eta }}_{v_1}^x{\boldsymbol{\eta }}_{v_2}^x{\boldsymbol{\eta }}_{v_3}^x + J_{\mathrm{m}}\mathop {\sum}\nolimits_p {\kern 1pt} {\boldsymbol{\eta }}_{p_1}^z \cdots {\boldsymbol{\eta }}_{p_6}^z + \cdots ,} \hfill \\ {} \hfill & \equiv \hfill & {J_{\mathrm{e}}\mathop {\sum}\nolimits_v {\kern 1pt} {\mathbf{A}}_v + J_{\mathrm{m}}\mathop {\sum}\nolimits_p {\kern 1pt} {\mathbf{B}}_p + \cdots } \hfill \end{array}$$

Here *v* and *p* label, respectively, the vertices and the plaquettes of the honeycomb superlattice, while the respective indices *v*_1_–*v*_3_ and *p*_1_–*p*_6_ are shown in Fig. 2b. In terms of the original lattice, the index *v* labels the non-empty hexagons of type *h*_*β*_, while *p* labels the empty hexagons of type *h*_*α*_. The omitted terms are the much weaker, higher-order terms, **B**_*p*_**B**_*p*′_, **A**_*v*_**A**_*v*′_, etc, discussed above.

The Toric code model corresponds to the two leading terms of Eq. (10). However, the remarkable properties of the Toric code are also present in the full model of Eq. (10). These properties stem from the relations ${\bf{A}}_v^2 = {\bf{B}}_p^2 = 1$ and the fact that $\left\{ {{\bf{A}}_v,{\bf{B}}_p,{\cal H}_{{\mathrm{eff}}}} \right\}$ is a set of mutually commuting operators^6^. This model is a *Z*_2_ lattice gauge theory^32, 33^, with the local gauge transformations generated by **A**_*v*_. In the following, we discuss the most important of these properties^6, 8, 10^.

### Topological sectors

On a torus, $\mathop {\prod}\nolimits_v {\kern 1pt} {\bf{A}}_v = \mathop {\prod}\nolimits_p {\kern 1pt} {\bf{B}}_p = 1$ and so there are *N*_*v*_ = 2^*N*/3−1^ and *N*_*p*_ = 2^*N*/6−1^ independent choices of **A**_*v*_ and **B**_*p*_, respectively, leading to 2^*N*/2−2^ states. So the quantum numbers {*A*_*v*_, *B*_*p*_} do not exhaust all 2^*N*/2^ states of ***η***’s. The missing quantum numbers are provided by the nonlocal operators ${\bf{X}}_1 = \mathop {\prod}\nolimits_{{\mathrm{C}}_{{\mathrm{X1}}}} {\kern 1pt} {\boldsymbol{\eta }}^x$ and ${\bf{X}}_2 = \mathop {\prod}\nolimits_{{\mathrm{C}}_{{\mathrm{X2}}}} {\kern 1pt} {\boldsymbol{\eta }}^x$, defined on the non-contractible loops C_X1_ and C_X2_ of Fig. 2b. These operators commute with **A**_*v*_ and **B**_*p*_, and with each other, and in addition ${\bf{X}}_1^2 = {\bf{X}}_2^2 = 1$. The quantum numbers {*A*_*v*_, *B*_*p*_, *X*_1_, *X*_2_} then exhaust the Hilbert space of ***η***’s.

### Ground states for *K* < 0

**Fig. 4:**
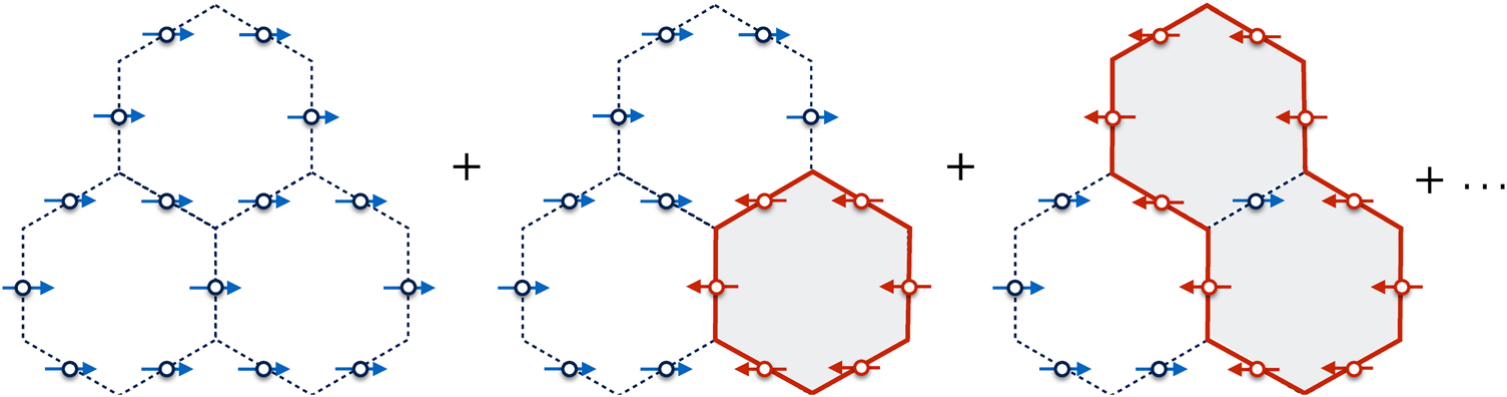
Pictorial representation of the quantum spin liquid ground states of the Toric code (10). These states are massive, equal-amplitude superpositions of all possible loops (red solid lines) of spins pointing along −**x** (red arrows), on top of a FM background of spins pointing along +**x** (blue arrows)

Without loss of generality, we will consider the *K* < 0 case, where both *J*_m_ and *J*_e_ are negative. Let us first ignore *J*_e_. As demonstrated below, the higher-order couplings between the fluxes are much weaker than $\left| {J_{\mathrm{m}}} \right|$, so the ground-state flux sector is the one with *B*_*p*_ = 1 for all *p*. This sector contains 2^*N*/3−1^ states, which are degenerate in the absence of *J*_e_, even when we include interactions between the fluxes. The tunneling term will lift the degeneracy and will select the states with *A*_*v*_ = 1 for all *v*. On a torus, there are four such states, which correspond to the choices of the winding numbers *X*_1_ and *X*_2_. One of them is

11 $$\left| {X_1 = 1,X_2 = 1} \right\rangle = {\cal N}\mathop {\prod}\nolimits_p \left( {1 + {\bf{B}}_p} \right)\left| {{\mathrm{FM}}_x} \right\rangle ,$$

where ${\cal N}$ is a normalization factor, and $\left| {{\mathrm{FM}}_x} \right\rangle = \left| { \to \cdots \to } \right\rangle$ is the fully polarized state along **x**, which has *A*_*v*_ = 1, ∀*v*. Expanding the product over (1 + **B**_*p*_) shows that this state is the equal-amplitude superposition of all possible loops of overturned spins (spins pointing along −**x**, which correspond to electric flux lines) on top of the FM background, see Fig. 4 and refs. ^7, 8^. The remaining three ground states of the Toric code, $\left| {X_1,X_2} \right\rangle = \left| { - 1,1} \right\rangle$, $\left| {1, - 1} \right\rangle$ and $\left| { - 1, - 1} \right\rangle$, arise by replacing the reference state $\left| {{\mathrm{FM}}_x} \right\rangle$ in Eq. (11) with ${\bf{Z}}_2\left| {{\mathrm{FM}}_x} \right\rangle$, ${\bf{Z}}_1\left| {{\mathrm{FM}}_x} \right\rangle$, and ${\bf{Z}}_1{\bf{Z}}_2\left| {{\mathrm{FM}}_x} \right\rangle$, respectively, where ${\bf{Z}}_1 = \mathop {\prod}\nolimits_{{\mathrm{C}}_{{\mathrm{Z1}}}} {\kern 1pt} {\boldsymbol{\eta }}^z$ and ${\bf{Z}}_2 = \mathop {\prod}\nolimits_{{\mathrm{C}}_{{\mathrm{Z2}}}} {\boldsymbol{\eta }}^z$, defined along C_Z1_ and C_Z2_ of Fig. 2b. These operators flip *X*_2_ and *X*_1_, respectively, because of the anti-commutation relations {**Z**_1_, **X**_2_} = 0 and {**Z**_2_, **X**_1_} = 0.

Note that the ground-state sector of the original Kitaev spin model is 12-fold and not 4-fold degenerate, because there are three ways to place the dimer pattern of Fig. 2a into the lattice and each sector has its own Toric code description.

### Excitations of ${\cal H}_{{\mathrm{eff}}}$ for *K* < 0 and deconfinement

The elementary excitations are pairs of static charges (vertices with *A*_*v*_ = −1), or pairs of static fluxes (plaquettes with *B*_*p*_ = −1). To an excellent approximation, their energy is given by ${\mathrm{\Delta }}_e \simeq 4\left| {J_{\mathrm{e}}} \right|$ and ${\mathrm{\Delta }}_m \simeq 4\left| {J_{\mathrm{m}}} \right|$, respectively. In particular, Δ_*m*_ scales roughly linearly with *S* (see Fig. 3), whereas Δ_*e*_ is exponentially small in *S*, as follows from Eq. (9), and practically vanishes for *S* ≥ 1 and realistic values of *K*.

**Fig. 5:**
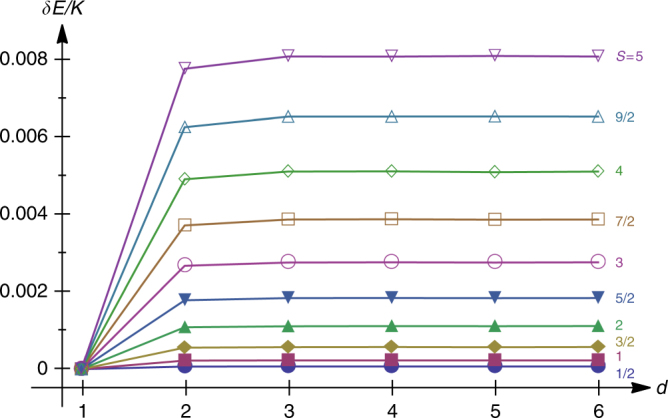
Magnetic flux deconfinement. Energy of a configuration with two magnetic fluxes as a function of the distance *d* between the associated empty hexagons, for spins *S* = 1/2 (bottom line) up to *S* = 5 (top line). The energy is measured from the value at nearest-neighbor separation *d* = 1. The data are extracted from exact diagonalizations on the mean-field decoupled Hamiltonian ${\cal H}_{{\mathrm{MF}}}$ discussed in the “Methods” section, using a 180-site honeycomb lattice on a torus, spanning an array of 15 × 2 empty hexagons. The data shown are along the horizontal direction up to *d* = 6 (higher *d* give a mirror image of the same data due to periodic boundary conditions)

Higher-order corrections to these expressions stem from the interactions between the respective pair of fluxes, which in particular depend on the distance between them. Figure 5 shows numerical data for the binding energy of two magnetic fluxes as a function of the distance *d* and the spin quantum number *S*. The data are extracted from an iterative exact diagonalization treatment of the mean-field decoupled Hamiltonian ${\cal H}_{{\mathrm{MF}}}$ described in the “Methods” section, taking a honeycomb lattice with 180 sites (i.e., 30 empty hexagon plaquettes) on a torus. The data show consistently that: first, the energy is lowest when the two fluxes reside next to each other; second, the energy levels off very quickly with the distance *d*, which demonstrates that the fluxes are deconfined; and third, the binding energy scale is at least one order of magnitude weaker than the leading *J*_m_ term (see Fig. 3), as announced already above.

### Origin of gauge structure and BSS fluxes

The local *Z*_2_ gauge symmetry of Eq. (10) is not an emergent property, but descends from the *Z*_2_ gauge structure of the original spin-*S* model, discovered by BSS^16^. This structure stems from the presence of local conserved operators defined on the hexagons of the original lattice, which are called BSS fluxes in the following. For the *h*_*β*_ hexagon of Fig. 2a, the BSS flux operator reads:

12 $${\bf{W}}_{{\mathrm{BSS}}}\left( {h_\beta } \right) = {\mathrm{exp}}\left[ {i\pi \left( {S_9^x + S_8^y + S_7^z + S_2^x + S_1^y + S_{10}^z} \right)} \right]{\kern 1pt} .$$

Now, the BSS fluxes on non-empty hexagons have the same effect as the **A**_*v*_ operators, e.g., ${\bf{W}}_{{\mathrm{BSS}}}\left( {h_\beta } \right)\left| {h_\beta } \right\rangle \to \left| {\bar h_\beta } \right\rangle$, modulo a numerical prefactor, see Eq. (13) below. So the local gauge symmetry of Eq. (10) indeed descends from that of the full model.

Let us now examine the ground-state BSS flux pattern. Unlike the original classical states associated with the “star” pattern, where only the empty hexagons have well-defined **W**_BSS_^16^, the QSL ground states of Eq. (10) have well-defined **W**_BSS_ on all hexagons. Indeed, using the same choice of local axes as the ones used above for the tunneling we find:

13 $$\left\langle {\bar h_\beta \left| {{\bf{W}}_{{\mathrm{BSS}}}\left( {h_\beta } \right)} \right|h_\beta } \right\rangle = \left( { - \kappa } \right)^{2S}{\kern 1pt} .$$

Now, the resonating QSL state $\left| {1,1} \right\rangle$ of Eq. (11) satisfies ${\mathrm{sgn}}\left( {J_{\mathrm{e}}} \right)A_v\left| {1,1} \right\rangle = - \left| {1,1} \right\rangle$, and therefore contains the combination $\frac{1}{{\sqrt 2 }}\left( {\left| {h_\beta } \right\rangle - {\mathrm{sgn}}\left( {J_{\mathrm{e}}} \right)\left| {\bar h_\beta } \right\rangle } \right)$. So, the ground-state expectation value *W*_BSS_(*h*_*β*_) of the operator **W**_BSS_(*h*_*β*_) is equal to

14 $$W_{{\mathrm{BSS}}}\left( {h_\beta } \right) = - ( - \kappa )^{2S + 1}{\kern 1pt} .$$

For half-integer *S*, in particular, *W*_BSS_(*h*_*β*_) = −1, irrespective of *κ*. For the empty hexagons, such as *h*_*α*_, a well-defined flux is already fixed by the zero-point energy, as shown by BSS^16^. Specifically, *W*_BSS_(*h*_*α*_) = (−1)^*λS*^, where *λ* = *κ*(*η*_1_ + *η*_3_ + *η*_5_) + *η*_2_ + *η*_4_ + *η*_6_, which is even. So, for integer *S*, *W*_BSS_(*h*_*α*_) = 1, while for half-integer *S*, ${W}_{{\mathrm{BSS}}}\left( {h_\alpha } \right) = - \kappa B_{h_\alpha } = - 1$, because of the ABC condition on spin waves.

The BSS fluxes are in fact well defined in all eigenstates of Eq. (10), not just in the ground states. An excited state with an electric charge sitting on *h*_*β*_ has *W*_BSS_(*h*_*β*_) = (−*κ*)^2*S*+1^, opposite to the one in the ground state. On the other hand, an excited state with a magnetic charge on *h*_*α*_ has *W*_BSS_(*h*_*α*_) = 1 for both integer and half-integer *S*. These results also mean that magnetic fluxes are related to BSS fluxes on empty hexagons for all *S*, and electric charges are related to BSS fluxes on non-empty hexagons for half-integer *S*.

More generally, the fact that the BSS fluxes are well defined on all hexagons is consistent with Elitzur’s theorem^34–36^ that local gauge symmetries cannot be broken spontaneously. Following the works in refs. ^16, 37^, this also necessitates that static and dynamic two-spin correlation functions are identically zero beyond NN separation, consistent with the Toric code description.

### Spin-wave modes

In the frozen dimer pattern of Fig. 2a, the local Hilbert space for each spin-*S* dimer has dimension (2*S* + 1)^2^, and Eq. (10) describes the dynamics inside the subspace of $\left| {m_1,m_2} \right\rangle = \left| {S,\kappa S} \right\rangle$ and $\left| { - S, - \kappa S} \right\rangle$, where the projections *m*_1_ and *m*_2_ are defined along the local quantization axes. To this Hamiltonian (10), we should also add the terms that describe the coherent spin-wave bosonic modes

15 $${\cal H}_{{\mathrm{magn}}}\left( {\left\{ {B_p} \right\}} \right) = \mathop {\sum}\nolimits_{i = 1}^N {\kern 1pt} \omega _i\left( {\left\{ {B_p} \right\}} \right) b_i^\dagger b_i,$$

describing the elementary, single-particle excursions outside this 2 × 2 manifold, with Δ*m* = ±1. Note that the important constants arising from the spin-wave theory have been assigned to *J*_m_ already, and that the *b*_*i*_ bosons are the eigenmodes of the spin-wave Hamiltonian, either at the quadratic or the self-consistent quartic order (see Supplementary Note 1). Also, as mentioned above, the spin-wave frequencies *ω*_*i*_ depend on the set {*B*_*p*_} only, and are therefore the same for all states with the same {*B*_*p*_} but different {*A*_*v*_}. This entails a huge, $2^{\frac{N}{3} - 1}$-fold degeneracy (in the torus geometry) in the spin-wave branches, for each given set of {*B*_*p*_}. We emphasize that the magnons discussed here do not describe the elementary excitations above some magnetically ordered state. Instead, they describe coherent excitations that are present in the spectrum independently of the elementary flux and charge excitations.

We now examine the actual structure of the magnon spectrum. At the quadratic level, BSS have shown^16^ that the spectrum consists of six flat bands, with *ω*_1–4_(**k**) = 0 and $\omega _{5,6}({\bf{k}}) = \sqrt 3 \left| K \right|S$, where the momentum *k* belongs to the magnetic Brillouin zone. However, the problem with the quadratic theory is that the modes 1–4 are not true zero modes, i.e., they will be gapped out by interactions. Such spurious zero modes are typical^17, 38–45^ artifacts of the harmonic theory and reflect the modes that connect different classical minima. As commented above, the large number of such spurious zero modes in the present model leads to unreliable estimates for the relevant energy scales of the problem. This necessitates that we push the semiclassical expansion to quartic order, and treat the problem via a standard self-consistent decoupling scheme (see Supplementary Note 1).

A key finding of this analysis is that spin waves remain localized inside the empty hexagons even at the interacting spin-wave level, because of the local conservation laws associated with the BSS fluxes. To see this, let us return to Eq. (6) and consider the interaction between different empty hexagons, *n*_1_*n*_10_, which is disregarded in the linear spin-wave theory. The standard mean-field decoupling of this term gives

16 $$n_1n_{10} \simeq p_1n_{10} + p_{10}n_1 + \left( {\delta {\kern 1pt} c_1c_{10} + m c_1c_{10}^\dagger + h.c.} \right) - \xi ,$$

where $\xi = p_1p_{10} + \left| \delta \right|^2 + \left| m \right|^2$, $p_i = \left\langle {n_i} \right\rangle$, $m = \left\langle {c_1^\dagger c_{10}} \right\rangle$, and $\delta = \left\langle {c_1^\dagger c_{10}^\dagger } \right\rangle$. Now, as discussed above, the states around which we expand do not break the BSS operators on empty hexagons. For the hexagon *h*_*α*_ of Fig. 2a, the BSS operator reads:

17 $${\mathbf{W}}_{{\mathrm{BSS}}}\left( {h_\alpha } \right) = e^{i\lambda S}{\kern 1pt} e^{i\pi \left[ {\kappa \left( {\eta _1n_1 + \eta _3n_3 + \eta _5n_5} \right) + \eta _2n_2 + \eta _4n_4 + \eta _6n_6} \right]},$$

where *λ* has been defined above. Hence, the invariance of the Hamiltonian and the state around which we expand under **W**_BSS_(*h*_*α*_) translates into the invariance of the parity of the number *κ*(*η*_1_*n*_1_ + *η*_3_*n*_3_ + *η*_5_*n*_5_) + *η*_2_*n*_2_ + *η*_4_*n*_4_ + *η*_6_*n*_6_. Since both *κ* and the ***η***-variables can only take the values +1 and −1, it follows that the parity of this number is the same as the parity of the total number of bosons, $N_{h_\alpha } = \mathop {\sum}\nolimits_{i = 1}^6 {\kern 1pt} n_i$, inside the hexagon *h*_*α*_. This means that terms that change the parity of $N_{h_\alpha }$ are not allowed in the expansion. As a result, the constants *m* and *δ* appearing in Eq. (16) vanish by symmetry, and magnons do not hop from one empty hexagon to another.

**Fig. 6:**
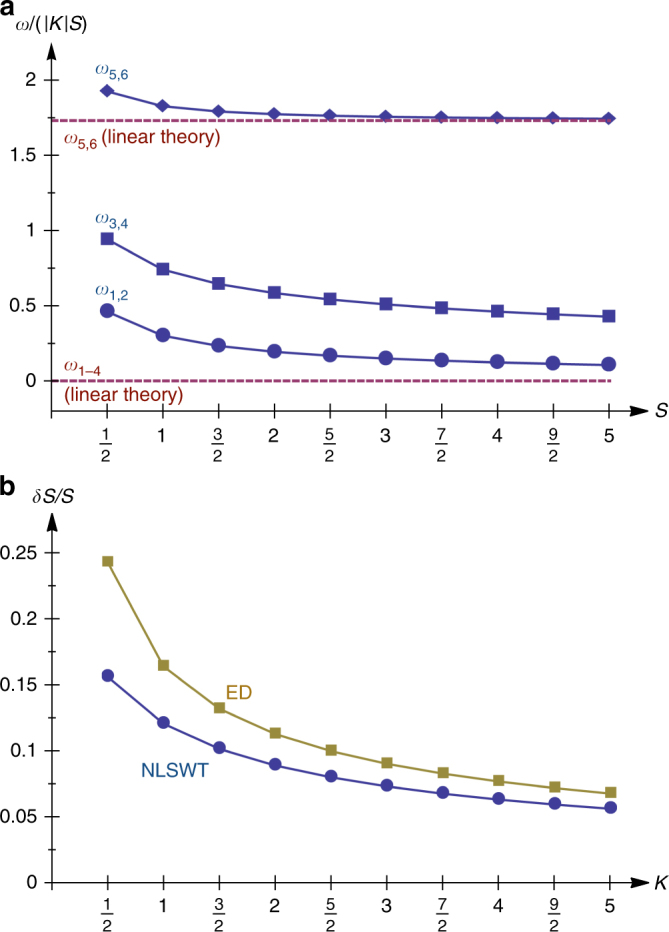
Spin-wave modes and relative spin-length correction. **a** Magnon frequencies *ω*_1_–*ω*_6_ (in units of $\left| K \right|S$) from linear (dashed)^16^ and non-linear spin-wave theory (solid), above the classical states associated with the dimer pattern of Fig. 2a, inside the uniform flux sector $B_{h_\alpha } = 1$ for all *h*_*α*_. **b** Relative spin length correction *δS*/*S* in the same sector, as extracted from non-linear spin-wave theory (NLSWT, blue circles) and exact diagonalizations (ED, dark yellow squares) of the Hamiltonian ${\cal H}_{{\mathrm{MF}}}$ discussed in the “Methods” section

Let us now focus on the excitations above the ground-state sector for *K* < 0, where *B*_*p*_ = 1 for all empty hexagons *p*. Given that the flux configuration is uniform, all constants $p_i = \left\langle {n_i} \right\rangle$ are equal and the interacting spin-wave problem then reduces to a self-consistent problem on a single hexagon, and in addition the magnon frequencies do not depend on the position of that hexagon. The calculated frequencies are shown in Fig. 6a along with the corresponding results from the quadratic theory. All spurious modes are gapped out, and the spectrum organizes into three degenerate pairs due to symmetry (see Supplementary Note 1). This figure also tells us that all modes sit far above the energy scales $\left| {J_{\mathrm{m}}} \right|$ and $\left| {J_{\mathrm{e}}} \right|$ of Eq. (10). In addition, the spin length corrections *δS* of Fig. 6b show that spin waves do not reduce the spin length appreciably (at maximum it is about 15% for *S* = 1/2), so the ***η*** variables are robust degrees of freedom.

### Physics at low *S*

**Fig. 7:**
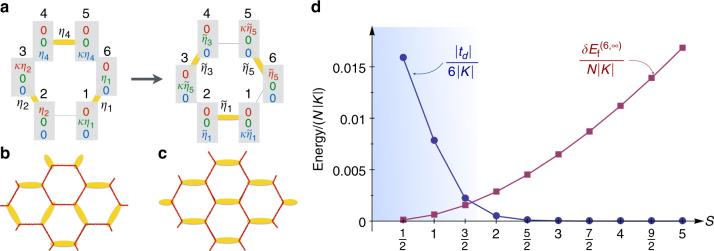
Breakdown of Toric code description at *S* ≲ 3/2. **a** The tunneling process that shifts the dimers around a hexagon, with a quantum mechanical amplitude *t*_D_. **b** The star dimer pattern of Fig. 2 (repeated here for comparison with **c**), where empty bonds form closed hexagons (red lines). **c** Dimer pattern where empty bonds form infinite strings (red lines). **d** Competition between kinetic energy (blue line, rescaled by a factor $6\left| K \right|$ to get the energy contribution per site, in units of $\left| K \right|$) and the dimer freezing energy *δE*^(6,∞)^ (red line, rescaled by $N\left| K \right|$ to get the energy contribution per site, in units of $\left| K \right|$). The latter is obtained by subtracting the potential energies (calculated at the interacting spin-wave level) of the two states depicted in **b**, **c**

We now turn our discussion to what can go wrong with the above semiclassical picture as we lower *S*. The dimer freezing in the star pattern of Fig. 2a stems from the zero-point energy of spin waves. However, this analysis disregards the quantum tunneling between different dimer patterns. The leading process is the one around a hexagon (Fig. 7a). The states associated with different dimer patterns are not orthonormal, but we can estimate the relevant tunneling amplitude *t*_*d*_ using the truncation method in ref. ^46^ (see “Methods”):

18 $$\left| {t_d} \right|{\mathrm{/}}\left| K \right| = 3S^22^{ - 6S}{\mathrm{/}}\left( {1 - 2^{ - 12S}} \right).$$

At large *S*, *t*_*d*_ is extremely small, and the spin-wave analysis of the dimer freezing has solid ground. This would in fact remain true down to *S* = 1, if we were to use linear spin-wave theory. However, this theory overestimates strongly the freezing energy scale (like $\left| {J_{\mathrm{m}}} \right|$) due to the spurious zero modes mentioned above. As a result, *t*_*d*_ becomes relevant below *S* ~ 3/2. To see this, let us take as a representative freezing energy scale, the energy difference $\delta E_{\mathrm{f}}^{(6,\infty )}$ between the star pattern (shown again in Fig. 7b for convenience) and the “staggered” pattern of Fig. 7c, where the empty loops have infinite length. At the level of interacting spin-wave theory, this energy difference is shown in Fig. 7d along with $\left| {t_d} \right|$ (where we divide by *N* and by 6, respectively, so that we compare energies per site). The results show clearly that dimers become mobile below *S* ~ 3/2. (By contrast, linear spin-wave theory gives $\delta E_{\mathrm{f}}^{(6,\infty )}{\mathrm{/}}(NK) = \left( {\frac{{\sqrt 3 }}{6} - \frac{1}{\pi }} \right)S$^16^, which is much larger than $\left| {t_d} \right|{\mathrm{/}}6$ down to *S* = 1.)

It follows that in order to understand the physics of the *S* = 3/2 and *S* = 1 cases, we need to return to the Cartesian basis, and allow both the position of the dimers and their spin orientation to resonate. Such a “decorated quantum dimer” description may appear quite more involved, but it may actually not be the case for the particular *S* = 1 case. The reason is that *t*_*d*_/6 is more than ten times larger than *δE*_f_/*N* for *S* = 1 (see Fig. 7d) and, from the standard quantum dimer model on the honeycomb lattice^28, 30^, we know that *t*_*d*_ stabilizes a resonating “plaquette” dimer pattern, known also from the context of the frustrated Heisenberg model^29, 47, 48^. Including the much smaller *J*_e_ term will include the resonances with the dimers of the opposite spin orientations. It would be interesting to check numerically this generalized semiclassical picture for *S* = 1, and moreover whether certain features of this picture carry over to the exactly solvable *S* = 1/2 case.

## Discussion

It is shown that the low-energy sector of the large-*S* Kitaev honeycomb model is described by a Toric code on a honeycomb superlattice. This should be contrasted with the effective square-lattice Toric code that arises in the spin-1/2 model when one of the three types of bonds has much stronger coupling than the other two^7^. Here, the magnetic and electric flux terms of the effective description arise respectively from the zero-point energy of spin waves and quantum mechanical tunneling between different orientations of frozen dimers. This picture breaks down for *S* ≲ 3/2, where tunneling between different dimer patterns becomes relevant.

The fundamental principle that prevents magnetic ordering in the semiclassical regime of the present model is the presence of an extensive number of local conservation laws, which were discovered in the seminal study by Baskaran, Sen and Shankar (BSS)^16^. According to Elitzur’s theorem^34^, these local symmetries cannot break spontaneously even at zero temperature, and the system fails to order magnetically even in the semiclassical limit. Given in addition that the conservation laws are not emergent, the gauge structure is not only present in the low-energy sector, but also in the single-particle, spin-wave channel, as we analyzed in detail beyond the quadratic level.

The prospects for realizing *S* > 1/2 Kitaev magnets remain at present limited, although there are reports for nearly perfect honeycomb magnets with Co^2+^ ions, such as Na_2_Co_2_TeO_6_ and Na_3_Co_2_SbO_6_^49^, with peculiar spatial magnetic correlations^50^. These systems show single-ion anisotropy, but it is worth checking via ab initio methods if a strong Kitaev term is also present, as in the layered spin-1/2 iridates and ruthenates^51–54^. In parallel, there are proposals for emulating the model with trapped ions^55^, superconducting quantum circuits^56^, coupled cavity arrays^57^, and ultracold atoms in optical lattices^58–61^, which in particular offer the possibility for *S* > 1/2 extensions of the model^59–61^.

Finally, we point out that the uniform^13^ or staggered^62, 63^ charge sectors of Eq. (10) describe another well known *Z*_2_ spin liquid, the RVB state of the spin-1/2 Heisenberg kagome antiferromagnet^46, 64–68^. This highlights the universal topological features of QSLs arising from very different settings, across both isotropic and highly anisotropic magnets.

## Methods

### Mean-field decoupled Hamiltonian ${\cal H}_{{\mathrm{MF}}}$

**Fig. 8:**
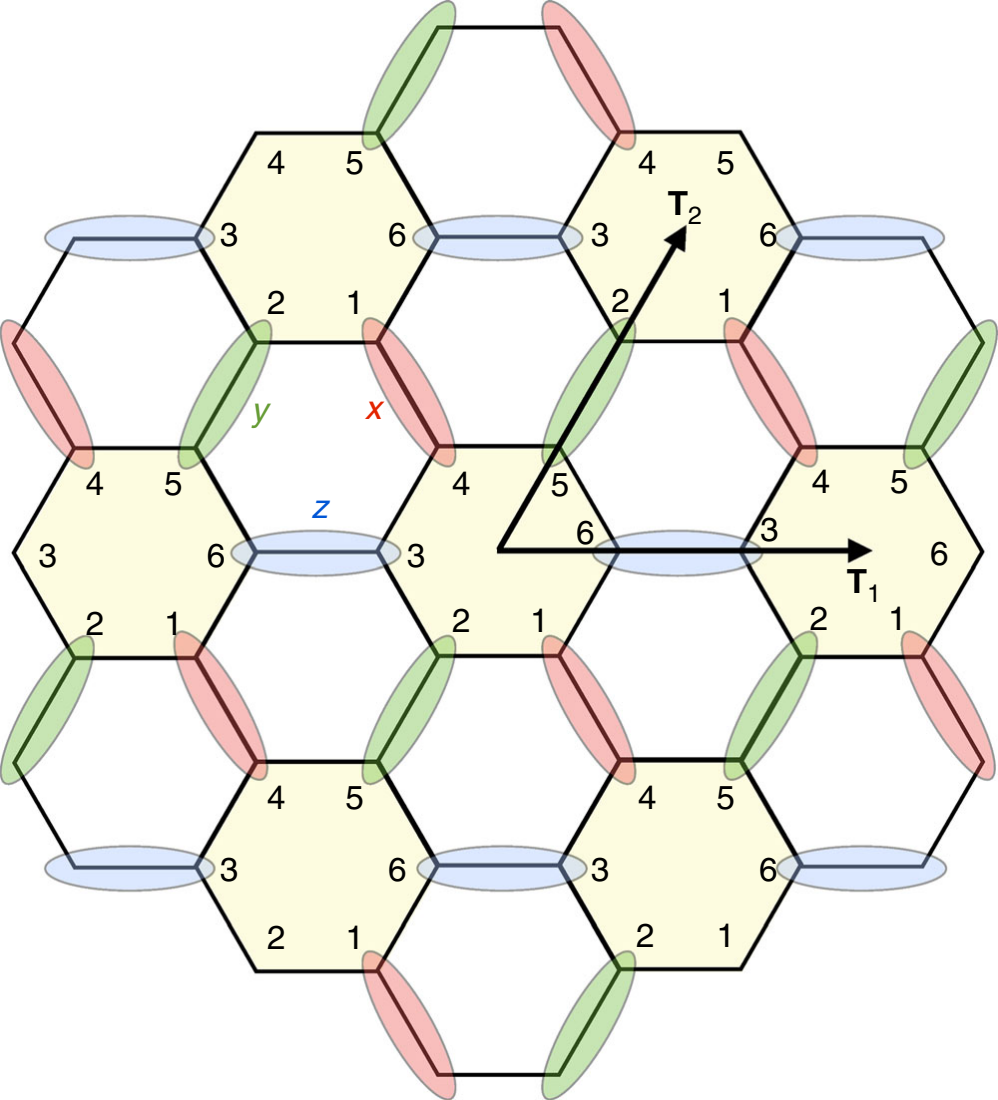
Six sublattice decomposition of the lattice corresponding to the star dimer pattern of Fig. 2. Each unit cell of the superlattice contains six spins, labeled by the numbers 1–6. The two black arrows show two primitive translations, **T**_1_ and **T**_2_, of this superlattice

Here, we discuss the exact diagonalization (ED) treatment that delivers the results shown in Figs. 3, 5, and 6b. Starting with the classical configurations of the star dimer pattern of Fig. 2, we introduce the six sublattice decomposition of Fig. 8, with a superlattice defined by the primitive translation vectors **T**_1_ and **T**_2_. The sites *i* of the lattice can then be labeled as *i* = (**R**, *ν*), where **R** is a primitive vector of the superlattice, and *ν* = 1–6 is the sublattice index. In this parametrization, the positions of the empty hexagons *h*_*α*_ are labeled by **R**. Each given classical state is parametrized in terms of the ***η***-variables, as shown in Fig. 2. With the local coordinate frames of Eq. (3), the Hamiltonian reads ${\cal H} = {\cal H}_0 + {\cal V}$, where ${\cal H}_0$ describes intra-hexagon terms,

19 $$\begin{array}{*{20}{l}} {{\cal H}_0} \hfill & = \hfill & {K\mathop {\sum}\limits_{\bf{R}} \left[ {S_{{\bf{R}},1}^uS_{{\bf{R}},2}^u + S_{{\bf{R}},2}^vS_{{\bf{R}},3}^v + S_{{\bf{R}},3}^uS_{{\bf{R}},4}^u} \right.} \hfill \\ {} \hfill & {} \hfill & {\left. { + S_{{\bf{R}},4}^vS_{{\bf{R}},5}^v + S_{{\bf{R}},5}^uS_{{\bf{R}},6}^u - \kappa B_RS_{{\bf{R}},6}^vS_{{\bf{R}},1}^v} \right],} \hfill \end{array}$$

and ${\cal V}$ accounts for the inter-hexagon terms,

20 $${\cal V} = - \left| K \right|\mathop {\sum}\limits_{\bf{R}} \left[ {S_{{\bf{R}},3}^wS_{{\bf{R}} - {\bf{T}}_1,6}^w + S_{{\bf{R}},1}^wS_{{\bf{R}} + T_3,4}^w + S_{{\bf{R}},5}^wS_{{\bf{R}} + {\bf{T}}_2,2}^w} \right],$$

where **T**_3_ = **T**_1_ − **T**_2_.

Now, the semiclassical analysis rests on the assumption that the expectation value of the operators $S_i^w$ is finite and relatively close to the classical value *S*. The spin-wave expansion, which has been discussed in the text and in the Supplementary Notes 1 and 2, is the standard way to proceed. Another way is to perform a mean-field decoupling of the inter-hexagon terms in ${\cal V}$, i.e., to replace

21 $$S_i^wS_j^w \to \left\langle {S_i^w} \right\rangle S_j^w + \left\langle {S_j^w} \right\rangle S_i^w - \left\langle {S_i^w} \right\rangle \left\langle {S_j^w} \right\rangle .$$

The resulting mean-field Hamiltonian ${\cal H}_{{\mathrm{MF}}}$ describes a collection of single hexagon Hamiltonians, each of which contains local Zeeman fields that depend on the state of the neighboring hexagons.

The ED results shown in Figs. 3, 5, and 6b are obtained using the following iterative procedure. We consider a honeycomb lattice on a torus with a certain number of empty hexagons. Then, we fix the numbers *κB*_**R**_ depending on the flux configuration we are interested in. In the next step, we initialize the spin lengths to their classical values *S*, the same for all sites. Then, we go through all empty hexagons, one after the other, and diagonalize numerically the corresponding Hamiltonian. From the ground state we then calculate the expectation values of the six spins of the hexagon $\left\langle {S_i^w} \right\rangle$, and then update the corresponding Zeeman terms for the neighboring hexagons. This iterative procedure converges very fast in energy, leading to a total energy of the system and the distribution of spin lengths. Note that for uniform flux configurations, such as the ground-state sector where *κB*_**R**_ = 1 for all **R**, the expectation values $\left\langle {S_i^w} \right\rangle$ are the same throughout the system, and we can therefore use a single hexagon only. For non-uniform flux configurations, on the other hand, the expectation values $\left\langle {S_i^w} \right\rangle$ are non-uniform as well, and we need to do a self-consistent calculation on a large enough lattice.

The ED results shown in Fig. 3 for the coupling *J*_m_ are obtained by comparing the energy of the ground-state flux sector, where *κB*_**R**_ = 1 for all **R**, with the energy of the sector with *κB*_**R**_ = −1 for all **R**. Strictly speaking, this method (called method 1 in Table 1) does not deliver the coupling *J*_m_, because the energy of the second sector contains contributions from the interactions between fluxes. A more accurate determination of *J*_m_ arises by comparing the energy of the ground-state sector with the energy of the sector with a single flux, i.e., the sector with *κB* equal to −1 at a single empty hexagon and +1 elsewhere. The last two rows of Table 1 show the numerical results for *J*_m_ obtained using this method (called method 2 in Table 1) on a periodic lattice with 84 sites (spanning an array of 7 × 2 empty hexagons) and another with 180 sites (spanning an array of 15 × 2 empty hexagons). For comparison, we also provide in the second row the results shown in Fig. 3 using the first method. The differences between the three last rows are tiny, showing once again that the interactions between fluxes are much weaker than *J*_m_.

**Table 1:** Numerical values of *J*_m_/*K* extracted from the non-linear spin-wave theory (first row), and exact diagonalizations (last three rows)

Spin *S*	1/2	1	3/2	2	5/2	3	7/2	4	9/2	5
NLSWT	0.000664	0.003722	0.009381	0.017415	0.027566	0.039597	0.053310	0.068535	0.085129	0.102969
ED (method 1)	0.002667	0.008068	0.016702	0.027916	0.041309	0.056579	0.073496	0.091869	0.111548	0.132408
ED (method 2–84 sites)	0.002765	0.008384	0.017487	0.029420	0.043764	0.060226	0.078561	0.098494	0.119947	0.142784
ED (method 2–180 sites)	0.002765	0.008384	0.017489	0.029418	0.043756	0.060212	0.078550	0.098457	0.119919	0.142785

The results of the second row are obtained by comparing the energy of the ground-state flux sector, where *κB*_*R*_ = 1 for all **R**, with the energy of the sector with *κB*_*R*_ = −1 for all **R**. By contrast, the results of the last two rows are obtained from tori clusters with 84 and 180 sites, by comparing, in each case, the energy of the ground-state sector with the energy of the sector with a single flux (i.e., the sector with *κB* equal to −1 at a single empty hexagon and +1 elsewhere)

The first row of Table 1 gives the corresponding results from non-linear spin-wave theory (NLSWT), which are shown in Fig. 3. These results are obtained by an iterative solution of the mean-field decoupled spin-wave Hamiltonian, by comparing the energy of the ground-state flux sector, where *κB*_**R**_ = 1 for all **R**, with the energy of the sector with *κB*_**R**_ = −1 for all **R**.

Next, the results for the binding energy of two fluxes that are shown in Fig. 5 are obtained by the iterative ED procedure described above on a periodic lattice with 180 sites, that spans an array of 15 × 2 empty hexagons.

Finally, the ED results for the spin length correction shown in Fig. 6b are obtained by the iterative ED procedure above the ground-state flux configuration, which reduces to an iterative procedure on a single hexagon as noted above.

### Comparison between ED and NLSWT

As we discussed in the main text, magnons do not hop from one empty hexagon to another even at the interacting spin-wave level, because of the local BSS conservation laws. As a result, the constants *m* and *δ* in Eq. (16) vanish by symmetry and the same is true for all similar terms arising in the lattice. Now, Eq. (16) becomes

22 $$n_1n_{10} \simeq p_1n_{10} + p_{10}n_1 - p_1p_{10}.$$

Written back in terms of spin operators, we recognize that this is the precisely the mean-field decoupling of $S_i^wS_j^w$ that leads to the Hamiltonian ${\cal H}_{{\mathrm{MF}}}$ discussed above. Essentially then, the NLSWT is a truncation of the Hamiltonian ${\cal H}_{{\mathrm{MF}}}$ using the bosonic representation of spins up to a given order in 1/*S*. So the iterative ED results are more accurate than the ones from the iterative NLSWT. The two methods should of course agree at large enough *S*, which is consistent with the trend shown in Figs. 3 and 6b.

### Derivation of Eq. (18)

To calculate the tunneling *t*_*d*_ around a single hexagon, we consider the simplest 2 × 2 truncation approach described in ref. ^46^ (see also ref. ^69^). Namely, we take a hexagon cluster and project the Hamiltonian into the 2 × 2 basis of dimer states shown in Fig. 7a:

23 $$\begin{array}{*{20}{l}} {\left| 1 \right\rangle = \left| {\kappa \eta _1{\bf{y}}} \right\rangle _1\left| {\eta _2{\bf{x}}} \right\rangle _2\left| {\kappa \eta _2{\bf{x}}} \right\rangle _3\left| {\eta _4{\bf{z}}} \right\rangle _4\left| {\kappa \eta _4{\bf{z}}} \right\rangle _5\left| {\eta _1{\bf{y}}} \right\rangle _6} \hfill \\ {\left| 2 \right\rangle = \left| {\kappa \tilde \eta _1{\bf{z}}} \right\rangle _1 \left| {\tilde \eta _1{\bf{z}}} \right\rangle _2\left| {\kappa \tilde \eta _3{\bf{y}}} \right\rangle _3\left| {\tilde \eta _3{\bf{y}}} \right\rangle _4\left| {\kappa \tilde \eta _5{\bf{x}}} \right\rangle _5\left| {\tilde \eta _5{\bf{x}}} \right\rangle _6} \hfill \end{array}.$$

The magnitude of the overlap Ω between the two states is

24 $$\left| {\mathrm{\Omega }} \right| = \left| {\left\langle {1|2} \right\rangle } \right| = 2^{ - 6S},$$

and the matrix elements of the cluster Hamiltonian are

25 $$\begin{array}{*{20}{c}} {E_0 \equiv \left\langle {1\left| {\cal H} \right|1} \right\rangle = \left\langle {2|{\cal H}|2} \right\rangle = - 3\left| K \right|S^2} \\ {v \equiv \left\langle {1|{\cal H}|2} \right\rangle = - 6\left| K \right|S^2{\mathrm{\Omega }}} \end{array}.$$

Orthonormalizing the basis leads to the effective 2 × 2 Hamiltonian $$\left( {\begin{array}{*{20}{c}} {E_0 + v} & {t_d} \\ {t_d} & {E_0 + v} \end{array}} \right)$$, where the tunneling amplitude *t*_*d*_ and the potential energy *V* are given by^46, 69^

26 $$t_d = \frac{{v - E_0{\mathrm{\Omega }}}}{{1 - {\mathrm{\Omega }}^2}} = - \frac{{3KS^22^{ - 6S}}}{{1 - 2^{ - 12S}}} \times {\mathrm{sgn}}({\mathrm{\Omega }}), V = - {\mathrm{\Omega }}t_d.$$

The latter is much smaller than $\left| {t_d} \right|$ and can be ignored.

### Data availability

The data that support the findings of this study as well as the numerical codes are available from the corresponding author on reasonable request.

## Electronic supplementary material

Supplementary Information
