# Supplementary material for: Quantum spin liquid in the semiclassical regime
Source: Nat Commun. 2018 Apr 23;9:1575. doi: 10.1038/s41467-018-03934-1 (PMC5913244; doi:10.1038/s41467-018-03934-1)
Supplement: Supplementary file 1 — Supplementary Information [file 41467_2018_3934_MOESM1_ESM.pdf]

Supplementary information

Supplementary Figures

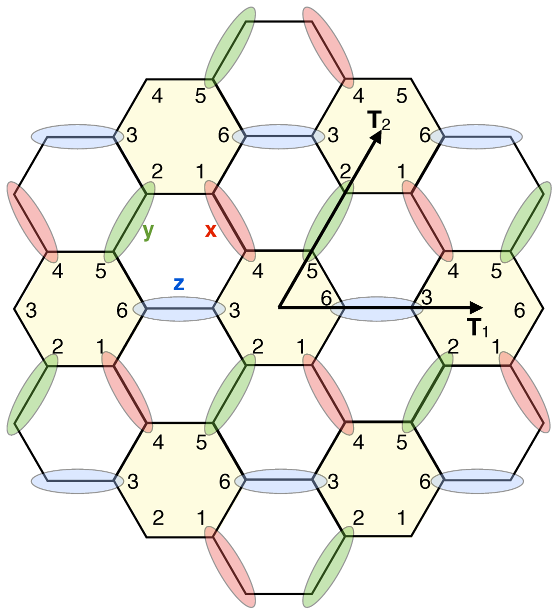

Supplementary Figure 1: The star dimer pattern of Fig. 2 of the main text and the six-sublattice decomposition used here. Each unit cell of the superlattice has 6 sites, labeled with the numbers 1-6. The vectors $\mathbf{T}_1$ and $\mathbf{T}_2$ are two primitive translations of the superlattice.

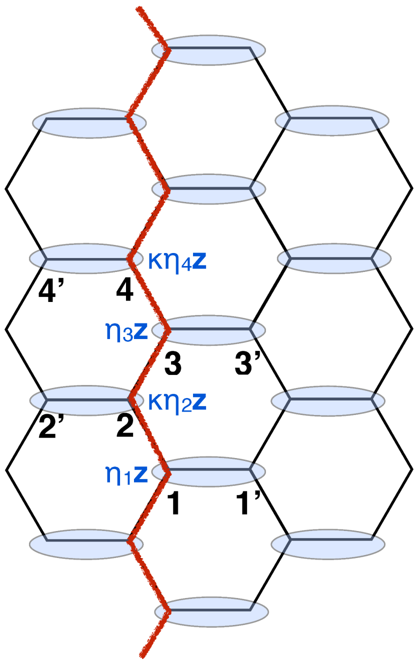

Supplementary Figure 2: Classical states associated with the staggered dimer pattern of Fig. 7 **c** of the main text. In this dimer pattern, the empty bonds form infinite strings (red, vertical zigzag line).

Supplementary Notes

Supplementary Note 1. Semiclassical expansion around the states of the star dimer pattern

Lattice superstructure & Hamiltonian

Here we provide the details of the semiclassical expansion around the states of the star dimer pattern of Fig. 2 of the main text. The analysis is based on the six-sublattice decomposition shown in Fig. 8 of the main text, but we repeat the details here for completeness. The six-sublattice decomposition is shown again in Supplementary Figure 1, with a superlattice defined by the primitive translation vectors $\mathbf{T}_1$ and $\mathbf{T}_2$. Any given site i of the lattice can be labeled as $i=(\mathbf{R},\nu)$, where $\mathbf{R}$ is a primitive vector of the superlattice and $\nu=$ 1-6 is the sublattice index. In this parametrization, the positions of the empty hexagons h_α are labeled by $\mathbf{R}$. The classical state is parametrized in terms of the η-variables, as shown in Fig. 2 of the main text. We will also use the local coordinate frames given in Eq. (3) of the main text, and define for each empty hexagon $h_{\mathbf{R}}$

$$\gamma_{\mathbf{R}} \equiv \kappa B_{\mathbf{R}}\,. \tag{1}$$

With these conventions and definitions, the Hamiltonian reads

$$\begin{aligned}\mathcal{H} &= K\sum_{\mathbf{R}}\left[S^u_{\mathbf{R},1}S^u_{\mathbf{R},2}+S^v_{\mathbf{R},2}S^v_{\mathbf{R},3}+S^u_{\mathbf{R},3}S^u_{\mathbf{R},4}+S^v_{\mathbf{R},4}S^v_{\mathbf{R},5}+S^u_{\mathbf{R},5}S^u_{\mathbf{R},6}-\gamma_{\mathbf{R}}S^v_{\mathbf{R},6}S^v_{\mathbf{R},1}\right]\\ &\quad -|K|\sum_{\mathbf{R}}\left[S^w_{\mathbf{R},3}S^w_{\mathbf{R}-\mathbf{T}_1,6}+S^w_{\mathbf{R},1}S^w_{\mathbf{R}+\mathbf{T}_1-\mathbf{T}_2,4}+S^w_{\mathbf{R},5}S^w_{\mathbf{R}+\mathbf{T}_2,2}\right]. \end{aligned}\tag{2}$$

Semiclassical expansion

In our semiclassical expansion we will keep up to four boson terms. So it suffices to keep the following terms from the standard [1] Holstein-Primakoff expansion for each site $i=(\mathbf{R},\nu)$:

$$\begin{aligned} S^w_i = S - c_i^+c_i,\;\; S_i^+ \simeq \sqrt{2S}(c_i - \tfrac{n_i}{4S}c_i),\;\; S_i^- \simeq \sqrt{2S}(c_i^+ - c_i^+\tfrac{n_i}{4S})\\ S^u_i \simeq \tfrac{\sqrt{S}}{\sqrt{2}}(c_i + c_i^+ - \tfrac{n_i}{4S}c_i - c_i^+\tfrac{n_i}{4S}),\;\; S^v_i \simeq -i\tfrac{\sqrt{S}}{\sqrt{2}}(c_i - c_i^+ - \tfrac{n_i}{4S}c_i + c_i^+\tfrac{n_i}{4S}) \end{aligned}\tag{3}$$

where c_i, c_i^+ are bosonic operators. We have:

$$\begin{aligned} S^u_iS^u_j \simeq \tfrac{S}{2}\Big(c_ic_j + c_ic_j^+ + h.c.\Big) - \tfrac{1}{8}\Big(c_in_jc_j + c_i^+n_jc_j + c_jn_ic_i + c_j^+n_ic_i + h.c.\Big)\\ S^v_iS^v_j \simeq -\tfrac{S}{2}\Big(c_ic_j - c_ic_j^+ + h.c.\Big) + \tfrac{1}{8}\Big(c_in_jc_j - c_i^+n_jc_j + c_jn_ic_i - c_j^+n_ic_i + h.c.\Big) \end{aligned}\tag{4}$$

Below we shall make use of the following mean-field parameters

$$\boxed{p_i = \langle c_i^+c_i\rangle},\quad \boxed{q_i=\langle c_ic_i\rangle},\quad \boxed{m_{ij}=\langle c_ic_j^+\rangle},\quad \boxed{\delta_{ij}=\langle c_ic_j\rangle}\,. \tag{5}$$

These parameters are all real numbers because when written in the local coordinate frames above, the Hamiltonian has real matrix elements, and in addition the states around which we expand are real. This also implies the relations $m_{ij}=m_{ji}$ and $\delta_{ij}=\delta_{ji}$. Next, we can decouple the quartic terms as follows:

$$\begin{aligned} c_in_jc_j \simeq m_{ij}c_jc_j + 2\delta_{ij}n_j + 2p_jc_ic_j + q_jc_ic_j^+ - m_{ij}q_j - 2\delta_{ij}p_j\\ c_i^+n_jc_j \simeq \delta_{ij}c_jc_j + 2m_{ij}n_j + 2p_jc_i^+c_j + q_jc_i^+c_j^+ - \delta_{ij}q_j - 2m_{ij}p_j \end{aligned}\tag{6}$$

Let us now write down the resulting expressions for each type of interaction that appears in the Hamiltonian.

- *Terms of the type $S_i^u S_j^u$ (where i and j belong to the same empty hexagon):*

$$\boxed{S_i^u S_j^u \simeq \tau_{ij} + \Big(f_{ji} c_j c_j + f_{ij} c_i c_i + g_{ij} c_i c_j + g_{ij} c_i c_j^+ + h.c.\Big) + 4 f_{ij}(n_j + n_i)} \tag{7}$$

where

$$\boxed{f_{ij} = -\frac{m_{ij}+\delta_{ij}}{8}, \quad g_{ij} = \frac{S}{2} - \frac{2(p_i+p_j)+q_i+q_j}{8}, \quad \tau_{ij} = -2f_{ij}[q_i+q_j+2(p_i+p_j)]} \tag{8}$$

- *Terms of the type $S_i^v S_j^v$ (where i and j belong to the same empty hexagon):*

$$\boxed{S_i^v S_j^v \simeq \tau'_{ij} + \Big(f'_{ij} c_j c_j + f'_{ij} c_i c_i + g'_{ij} c_i c_j - g'_{ij} c_i c_j^+ + h.c.\Big) - 4 f'_{ij}(n_i + n_j)} \tag{9}$$

where

$$\boxed{f'_{ij} = \frac{m_{ij}-\delta_{ij}}{8}, \quad g'_{ij} = -\frac{S}{2} + \frac{2(p_i+p_j)-q_i-q_j}{8}, \quad \tau'_{ij} = -2f'_{ij}[q_i+q_j-2(p_i+p_j)]} \tag{10}$$

- *Terms of the type $S^w_{\mathbf{R},\nu} S^w_{\mathbf{R}+\mathbf{T}_{\nu\mu},\mu}$:* The terms that couple different empty hexagons are of the form $S^w_{\mathbf{R},\nu} S^w_{\mathbf{R}+\mathbf{T}_{\nu\mu},\mu}$. For simplicity, we will label $(\mathbf{R},\nu) \to i$ and $(\mathbf{R}+\mathbf{T}_{\nu\mu},\mu) \to j$. We have:

$$S_i^w S_j^w = \big(S - n_i\big)\big(S - n_j\big) = \big(S^2 - S(n_i + n_j) + n_i n_j\big)\,. \tag{11}$$

The quartic term decouples as follows:

$$n_i n_j \quad \simeq \quad \big(p_j n_i + p_i n_j\big) + \big(\delta_{ij} c_i c_j + h.c.\big) + \big(m_{ij} c_i c_j^+ + h.c\big) - p_i p_j - \delta_{ij}^2 - m_{ij}^2\,. \tag{12}$$

We now repeat the main arguments described in the Methods section of the main paper that simplify this expression. The state around which we expand does not break the local BSS flux operators defined on the empty hexagons:

$$\begin{aligned} W_{\text{BSS}}(\mathbf{R}) &= \exp\big\{i\pi\big[S^x_{\mathbf{R},\mathbf{1}} + S^y_{\mathbf{R},\mathbf{2}} + S^z_{\mathbf{R},\mathbf{3}} + S^x_{\mathbf{R},\mathbf{4}} + S^y_{\mathbf{R},\mathbf{5}} + S^z_{\mathbf{R},\mathbf{6}}\big]\big\} \\ &= \exp\big\{i\pi\big[\kappa\big(\eta_1 S^w_{\mathbf{R},\mathbf{1}} + \eta_3 S^w_{\mathbf{R},\mathbf{3}} + \eta_5 S^w_{\mathbf{R},\mathbf{5}}\big) + \big(\eta_2 S^w_{\mathbf{R},\mathbf{2}} + \eta_4 S^w_{\mathbf{R},\mathbf{4}} + \eta_6 S^w_{\mathbf{R},\mathbf{6}}\big)\big]\big\} \\ &= (-1)^{\lambda_{\mathbf{R}} S} \exp\big\{-i\pi\big[\kappa\big(\eta_1 n_{\mathbf{R},\mathbf{1}} + \eta_3 n_{\mathbf{R},\mathbf{3}} + \eta_5 n_{\mathbf{R},\mathbf{5}}\big) + \big(\eta_2 n_{\mathbf{R},\mathbf{2}} + \eta_4 n_{\mathbf{R},\mathbf{4}} + \eta_6 n_{\mathbf{R},\mathbf{6}}\big)\big]\big\}\,, \end{aligned} \tag{13}$$

where $n_i = c_i^+ c_i$ is the boson number operator and $\lambda_{\mathbf{R}} = \kappa(\eta_{\mathbf{R},1} + \eta_{\mathbf{R},3} + \eta_{\mathbf{R},5}) + (\eta_{\mathbf{R},2} + \eta_{\mathbf{R},4} + \eta_{\mathbf{R},6})$, see main text. The invariance of the Hamiltonian and the state around which we expand under this operation translates into the invariance of the parity of the number $\kappa\big(\eta_1 n_{\mathbf{R},\mathbf{1}} + \eta_3 n_{\mathbf{R},\mathbf{3}} + \eta_5 n_{\mathbf{R},\mathbf{5}}\big) + \big(\eta_2 n_{\mathbf{R},\mathbf{2}} + \eta_4 n_{\mathbf{R},\mathbf{4}} + \eta_6 n_{\mathbf{R},\mathbf{6}}\big)$. But since κ and η can only take the values $+1$ and -1, it follows that the parity of this number is the same as the parity of the total number $N_{\mathbf{R}}$ of bosons in any given empty hexagon:

$$N_{\mathbf{R}} = \sum_{\nu=1-6} n_{\mathbf{R},\nu}\,. \tag{14}$$

This means that terms that change the parity of $N_{\mathbf{R}}$ are not allowed in the expansion. This excludes terms of the type $c_i c_j$ or $c_i c_j^+$, where i and j belong to different empty hexagons (see definition above). Equivalently, the mean-field parameters m_{ij} and δ_{ij} vanish by symmetry, and this is true to all orders in the Holstein-Primakoff expansion. We therefore get:

$$S_i^w S_j^w \simeq \Big\{\frac{S^2 - p_i p_j}{2} + (p_j - S) n_i\Big\} + \Big\{\frac{S^2 - p_i p_j}{2} + (p_i - S) n_j\Big\}\,, \tag{15}$$

i.e. empty hexagons decouple from each other and the $S_i^w S_j^w$ terms give, for each empty hexagon $\mathbf{R}$ alone, a

contribution

$$\boxed{\frac{S^2 - p_i p_j}{2} + (p_j - S) n_i} \tag{16}$$

where the constant $p_j = p_{\mathbf{R}+\mathbf{T}_{\nu\mu},\mu}$ refers to a neighboring hexagon and has to be found self-consistently in the general case.

It is useful to add here one more consequence of the BSS flux conservation. In the classical, reference state, where all n_i vanish, the BSS fluxes are equal to $(-1)^{\lambda_{\mathbf{R}} S}$ (see main text and [2]). Spin wave fluctuations dress the reference state but cannot change the BSS fluxes, because these are integer numbers. (13) then implies that the dressed ground state contains only terms with an even number of bosons $N_{\mathbf{R}}$.

Semiclassical expansion around the states of the star dimer pattern with uniform $\gamma_{\mathbf{R}}$

In the following we shall focus on the classical states that have the same $\gamma_{\mathbf{R}}$ on all empty hexagons. This means that $p_{\mathbf{R},\nu}$ is independent of $\mathbf{R}$, and we can therefore replace $p_{\mathbf{R}+\mathbf{T}_{\nu\mu},\mu} \to p_\mu$ in the above contribution from the $S_i^w S_j^w$ terms. Collecting all terms referring to a given hexagon and dropping the index $\mathbf{R}$ we get:

$$\mathcal{H}/|K| = f_0 + \frac{1}{2}\sum_\nu \Big\{ d_\nu c_\nu^+ c_\nu + d'_\nu c_\nu c_\nu + \lambda_{\nu,\nu+1} c_\nu c_{\nu+1} + \lambda_{\nu+1,\nu} c_{\nu+1} c_\nu + \pi_{\nu,\nu+1} c_\nu c_{\nu+1}^+ + h.c. \Big\}, \tag{17}$$

where we have defined:

$$\begin{aligned}
&f_0 = -3S^2 + \big(p_1 p_4 + p_2 p_5 + p_3 p_6\big) - \kappa\big(\tau_{12} + \tau_{34} + \tau_{56} + \tau'_{23} + \tau'_{45} - \gamma \tau'_{61}\big) - \tfrac{1}{2}\textstyle\sum_\nu d_\nu\,, \\
&d_1 = S - p_4 - 4\kappa\,(f_{12} + \gamma f'_{61}), \quad d'_1 = -2\kappa(f_{12} - \gamma f'_{16}), \quad \lambda_{12} = -\kappa g_{12}, \quad \pi_{12} = \lambda_{12} \\
&d_2 = S - p_5 - 4\kappa\,(f_{12} - f'_{23}), \quad d'_2 = -2\kappa(f_{21} + f'_{23}), \quad \lambda_{23} = -\kappa g'_{23}, \quad \pi_{23} = -\lambda_{23} \\
&d_3 = S - p_6 - 4\kappa\,(f_{34} - f'_{23}), \quad d'_3 = -2\kappa(f_{34} + f'_{32}), \quad \lambda_{34} = -\kappa g_{34}, \quad \pi_{34} = \lambda_{34} \\
&d_4 = S - p_1 - 4\kappa\,(f_{34} - f'_{45}), \quad d'_4 = -2\kappa(f_{43} + f'_{45}), \quad \lambda_{45} = -\kappa g'_{45}, \quad \pi_{45} = -\lambda_{45} \\
&d_5 = S - p_2 - 4\kappa\,(f_{56} - f'_{45}), \quad d'_5 = -2\kappa(f_{56} + f'_{54}), \quad \lambda_{56} = -\kappa g_{56}, \quad \pi_{56} = \lambda_{56} \\
&d_6 = S - p_3 - 4\kappa\,(f_{56} + \gamma f'_{61}), \quad d'_6 = -2\kappa(f_{65} - \gamma f'_{61}), \quad \lambda_{61} = \gamma\kappa g'_{61}, \quad \pi_{61} = -\lambda_{61}\,.
\end{aligned} \tag{18}$$

Next we define $\mathbf{C}^+ = \big(c_1^+, \cdots c_6^+, c_1, \cdots c_6\big)$ and write:

$$\mathcal{H} \;=\; f_0 + \frac{1}{2}\mathbf{C}^+ \cdot \mathbf{M} \cdot \mathbf{C}\,, \tag{19}$$

where the nonzero matrix elements of the matrix $\mathbf{M}$ are as follows:

$$\mathbf{M} = \left(\begin{array}{cccccc|cccccc}
d_1 & \pi_{12} & & & & \pi_{61} & d'_1 & \lambda_{12} & & & & \lambda_{61} \\
\pi_{12} & d_2 & \pi_{23} & & & & \lambda_{12} & d'_2 & \lambda_{23} & & & \\
& \pi_{23} & d_3 & \pi_{34} & & & & \lambda_{23} & d'_3 & \lambda_{34} & & \\
& & \pi_{34} & d_4 & \pi_{45} & & & & \lambda_{34} & d'_4 & \lambda_{45} & \\
& & & \pi_{45} & d_5 & \pi_{56} & & & & \lambda_{45} & d'_5 & \lambda_{56} \\
\pi_{61} & & & & \pi_{56} & d_6 & \lambda_{61} & & & & \lambda_{56} & d'_6 \\
\hline
d'_1 & \lambda_{12} & & & & \lambda_{61} & d_1 & \pi_{12} & & & & \pi_{61} \\
\lambda_{12} & d'_2 & \lambda_{23} & & & & \pi_{12} & d_2 & \pi_{23} & & & \\
& \lambda_{23} & d'_3 & \lambda_{34} & & & & \pi_{23} & d_3 & \pi_{34} & & \\
& & \lambda_{34} & d'_4 & \lambda_{45} & & & & \pi_{34} & d_4 & \pi_{45} & \\
& & & \lambda_{45} & d'_5 & \lambda_{56} & & & & \pi_{45} & d_5 & \pi_{56} \\
\lambda_{61} & & & & \lambda_{56} & d'_6 & \pi_{61} & & & & \pi_{56} & d_6
\end{array}\right) \tag{20}$$

We next define the commutator matrix

$$\mathbf{g}=\mathbf{C}\cdot\mathbf{C}^\dagger - \left((\mathbf{C}^\dagger)^T\cdot\mathbf{C}^T\right)^T = \left(\begin{array}{c|c} \mathbb{1}_6 & 0 \\ \hline 0 & -\mathbb{1}_6 \end{array}\right), \tag{21}$$

where $\mathbb{1}_6$ stands for the identity 6×6 matrix, and then perform a standard [3] Bogoliubov transformation $\mathbf{C}=\mathbf{S}\cdot\mathbf{B}$, which must conserve the commutation relations $\tilde{\mathbf{g}}=\mathbf{g}$. This relation gives

$$\mathbf{S}^\dagger\cdot\mathbf{g}\cdot\mathbf{S}=\mathbf{g}\Rightarrow\mathbf{S}^{-1}=\mathbf{g}\cdot\mathbf{S}^\dagger\cdot\mathbf{g}\,. \tag{22}$$

The matrix $\mathbf{S}$ must also satisfy the relation

$$\mathbf{L}\cdot\mathbf{S}\cdot\mathbf{L}=\mathbf{S}^*, \quad \text{where} \quad \mathbf{L}=\begin{pmatrix} 0 & \mathbb{1}_6 \\ \mathbb{1}_6 & 0 \end{pmatrix}, \tag{23}$$

and at the same time diagonalize the Hamiltonian:

$$\mathcal{H}=f_0+\frac{1}{2}\mathbf{B}^\dagger\cdot(\mathbf{S}^\dagger\mathbf{M}\mathbf{S})\cdot\mathbf{B}=f_0+\frac{1}{2}\mathbf{B}^\dagger\cdot\mathbf{\Omega}_M\cdot\mathbf{B}, \tag{24}$$

where $\mathbf{\Omega}_M$ is diagonal and can be found from the eigenvalue equation $(\mathbf{gM})\cdot\mathbf{S}=\mathbf{S}\cdot(\mathbf{g}\mathbf{\Omega}_M)\equiv\mathbf{S}\cdot\mathbf{\Omega}_{gM}$. It can be shown [3] that the eigenvalues of $\mathbf{g}\cdot\mathbf{M}$ come in pairs $(\omega_\nu, -\omega_\nu)$, where $\nu=$ 1-6. We finally get

$$\mathcal{H}=f_0+\sum_{\nu=1-6}\omega_\nu\left(b_\nu^+b_\nu+\frac{1}{2}\right)\,. \tag{25}$$

The ground state energy is, in particular, given by $E_0=f_0+\frac{1}{2}\sum_\nu\omega_\nu$.

Mean field parameters: General relations

Let us define the six eigenvectors of the matrix $\mathbf{g}\cdot\mathbf{M}$ that correspond to non-negative eigenvalues by $\mathbf{X}_\nu$, $\nu=$ 1-6. Using:

$$c_i=\sum_{j=1-6}\left(S_{i,j}b_j+S_{i,6+j}b_j^+\right), \quad c_i^+=\sum_{j=1-6}\left(S_{6+i,j}b_j+S_{6+i,6+j}b_j^+\right)=\sum_{j=1-6}\left(S^*_{i,j+6}b_j+S^*_{i,j}b_j^+\right), \tag{26}$$

we get the following expressions for the mean-field parameters:

$$\begin{aligned} n_i &= \langle c_i^+c_i\rangle=\textstyle\sum_{\nu=1-6}|S_{i,6+\nu}|^2=\sum_\nu|S_{i+6,\nu}|^2=\sum_\nu|X_\nu(i+6)|^2\,,\\ \delta_{ij} &= \langle c_ic_j\rangle=\textstyle\sum_{\nu=1-6}S_{i,\nu}S_{j,6+\nu}=\sum_\nu S_{i,\nu}S^*_{j+6,\nu}=\sum_\nu X_\nu(i)X^*_\nu(j+6)\,,\\ m_{ij} &= \langle c_ic_j^+\rangle=\textstyle\sum_{\nu=1-6}S_{i,\nu}S_{j+6,\nu+6}=\sum_\nu S_{i,\nu}S^*_{j,\nu}=\sum_\nu X_\nu(i)X^*_\nu(j)\,, \end{aligned} \tag{27}$$

where $\mathbf{X}_\nu$ denotes the ν-th eigenvector of $\mathbf{g}\cdot\mathbf{M}$. Note that the last expressions in each line do not depend on the arbitrary phase for the eigenvectors $\mathbf{X}_\nu$, which come out arbitrary when we diagonalize the matrix $\mathbf{g}\cdot\mathbf{M}$ numerically.

Mean field parameters: Symmetry constraints

We have already mentioned that all mean-field parameters defined above are real quantities. Here we give a list of symmetry operations (of the Hamiltonian and of the classical state around which we expand) which reduce strongly the number of independent mean-field parameters.

- *Symmetry* $\mathbf{\Sigma}_1$. This is a π-rotation in real space around the center of the hexagon, followed by $\frac{\pi}{2}$-rotations around the local $\mathbf{w}$-axes in spin space:

$$\begin{aligned}&(S_1^u,S_1^v,S_1^w)\to(S_4^v,-S_4^u,S_4^w),\quad(S_2^u,S_2^v,S_2^w)\to(S_5^v,-S_5^u,S_5^w),\quad(S_3^u,S_3^v,S_3^w)\to(S_6^v,-S_6^u,S_6^w),\\&(S_4^u,S_4^v,S_4^w)\to(-\gamma S_1^v,\gamma S_1^u,S_1^w),\quad(S_5^u,S_5^v,S_5^w)\to(-\gamma S_2^v,\gamma S_2^u,S_2^w),\quad(S_6^u,S_6^v,S_6^w)\to(-\gamma S_3^v,\gamma S_3^u,S_3^w)\,.\end{aligned}\tag{28}$$

These relations are equivalent with

$$\boxed{c_1\to -ic_4,\;\; c_2\to -ic_5,\;\; c_3\to -ic_6,\;\; c_4\to +i\gamma c_1,\;\; c_5\to +i\gamma c_2,\;\; c_6\to +i\gamma c_3}\tag{29}$$

- *Symmetry* $\mathbf{\Sigma}_2$. This is a reflection through the bonds (3,6) in real space, followed by $\frac{\pi}{2}$-rotations around the local $\mathbf{w}$-axes in spin space:

$$\begin{aligned}&(S_1^u,S_1^v,S_1^w)\to(\gamma S_5^v,-\gamma S_5^u,S_5^w),\quad(S_2^u,S_2^v,S_2^w)\to(\gamma S_4^v,-\gamma S_4^u,S_4^w),\quad(S_3^u,S_3^v,S_3^w)\to(\gamma S_3^v,-\gamma S_3^u,S_3^w),\\&(S_4^u,S_4^v,S_4^w)\to(\gamma S_2^v,-\gamma S_2^u,S_2^w),\quad(S_5^u,S_5^v,S_5^w)\to(\gamma S_1^v,-\gamma S_1^u,S_1^w),\quad(S_6^u,S_6^v,S_6^w)\to(-S_6^v,S_6^u,S_6^w)\,.\end{aligned}\tag{30}$$

These relations are equivalent with:

$$\boxed{c_1\to -i\gamma c_5,\;\; c_2\to -i\gamma c_4,\;\; c_3\to -i\gamma c_3,\;\; c_4\to -i\gamma c_2,\;\; c_5\to -i\gamma c_1,\;\; c_6\to +ic_6}\tag{31}$$

- *Symmetry* $\mathbf{\Sigma}_3$. This is a reflection through the middle of the bonds (1,2) and (4,5) in real space, followed by zero or π-rotations around the local-$\mathbf{w}$ axes in spin space:

$$\begin{aligned}&(S_1^u,S_1^v,S_1^w)\to(-\gamma S_2^u,-\gamma S_2^v,S_2^w),\quad(S_2^u,S_2^v,S_2^w)\to(-\gamma S_1^u,-\gamma S_1^v,S_1^w),\quad(S_3^u,S_3^v,S_3^w)\to(S_6^u,S_6^v,S_6^w),\\&(S_4^u,S_4^v,S_4^w)\to(S_5^u,S_5^v,S_5^w),\quad(S_5^u,S_5^v,S_5^w)\to(S_4^u,S_4^v,S_4^w),\quad(S_6^u,S_6^v,S_6^w)\to(S_3^u,S_3^v,S_3^w)\,.\end{aligned}\tag{32}$$

These relations are equivalent with:

$$\boxed{c_1\to -\gamma c_2,\;\; c_2\to -\gamma c_1,\;\; c_3\to c_6,\;\; c_4\to c_5,\;\; c_5\to c_4,\;\; c_6\to c_3}\tag{33}$$

- *Symmetry* $\mathbf{\Sigma}_4$. This is a reflection through the bonds (1,4) in real space, followed by $\frac{\pi}{2}$-rotations around the local $\mathbf{w}$-axes in spin space:

$$\begin{aligned}&(S_1^u,S_1^v,S_1^w)\to(-\gamma S_1^v,\gamma S_1^u,S_1^w),\quad(S_2^u,S_2^v,S_2^w)\to(S_6^v,-S_6^u,S_6^w),\quad(S_3^u,S_3^v,S_3^w)\to(S_5^v,-S_5^u,S_5^w),\\&(S_4^u,S_4^v,S_4^w)\to(S_4^v,-S_4^u,S_4^w),\quad(S_5^u,S_5^v,S_5^w)\to(S_3^v,-S_3^u,S_3^w),\quad(S_6^u,S_6^v,S_6^w)\to(S_2^v,-S_2^u,S_2^w)\,.\end{aligned}\tag{34}$$

These relations are equivalent with:

$$\boxed{c_1\to +i\gamma c_1,\;\; c_2\to -ic_6,\;\; c_3\to -ic_5,\;\; c_4\to -ic_4,\;\; c_5\to -ic_3,\;\; c_6\to -ic_2}\tag{35}$$

- *Symmetry* $\mathbf{\Sigma}_5$. This is a $\pi/6$-rotation in real space, followed by a $\frac{\pi}{2}$-rotation around the local $\mathbf{w}$-axes in spin space:

$$\begin{aligned}&(S_1^u,S_1^v,S_1^w)\to(S_2^v,-S_2^u,S_2^w),\quad(S_2^u,S_2^v,S_2^w)\to(S_3^v,-S_3^u,S_3^w),\quad(S_3^u,S_3^v,S_3^w)\to(S_4^v,-S_4^u,S_4^w),\\&(S_4^u,S_4^v,S_4^w)\to(S_5^v,-S_5^u,S_5^w),\quad(S_5^u,S_5^v,S_5^w)\to(S_6^v,-S_6^u,S_6^w),\quad(S_6^u,S_6^v,S_6^w)\to(-\gamma S_1^v,\gamma S_1^u,S_1^w)\,.\end{aligned}\tag{36}$$

These relations are equivalent with:

$$\boxed{c_1\to -ic_2,\;\; c_2\to -ic_3,\;\; c_3\to -ic_4,\;\; c_4\to -ic_5,\;\; c_5\to -ic_6,\;\; c_6\to +i\gamma c_1}\tag{37}$$

Combining $\mathbf{\Sigma}_1$-$\mathbf{\Sigma}_5$ gives the following constraints for the mean-field parameters:

$$\boxed{\begin{array}{c}\forall\nu: \;\; q_\nu = 0, \;\; p_\nu = p, \\ \delta_{12} = -\delta_{23} = \delta_{34} = -\delta_{45} = \delta_{56} = \gamma\delta_{61}, \\ m_{12} = m_{23} = m_{34} = m_{45} = m_{56} = -\gamma m_{61} \equiv m\end{array}} \tag{38}$$

The mean field parameter m

The numerical, self-consistent treatment of the decoupled spin-wave Hamiltonian gives a vanishing mean-field parameter m. This result does not arise from symmetry and is true only in the asymptotic large-S limit. For general S, m is a very small number. To see this we consider the self-consistent mean-field Hamiltonian for a single hexagon, that corresponds to the decoupled semiclassical problem that we are dealing with:

$$\mathcal{H}_{\text{MF}} = -h_{\text{loc}}(S_1^w + S_2^w + S_3^w + S_4^w + S_5^w + S_6^w) + (S_1^u S_2^u + S_2^v S_3^v + S_3^u S_4^u + S_4^v S_5^v + S_5^u S_6^u - \gamma S_6^v S_1^v) \equiv -h_{\text{loc}} S_{\text{tot}}^w + \mathcal{V}\,, \tag{39}$$

where h_{loc} is the self-consistent field exerted from neighboring hexagons and we have taken $K = 1$ without loss of generality. In what follows we shall use the Néel operator $\mathcal{L}$ defined as

$$\mathcal{L} = S_1^w - S_2^w + S_3^w - S_4^w + S_5^w - S_6^w\,, \tag{40}$$

and the relations

$$[S_1^+ S_2^-, \mathcal{L}] = [S_1^+ S_2^-, S_1^w - S_2^w] = -2S_1^+ S_2^- \Rightarrow \langle g|[S_1^+ S_2^-, \mathcal{L}]|g\rangle = -2\langle g|S_1^+ S_2^-|g\rangle\,, \tag{41}$$

- For $S = 1/2$, the numerical, self-consistent solution gives $h_{\text{loc}} = 0.37888$ and $m = 0$. However, this relation is special to $S = 1/2$ because the self-consistent ground state $|g\rangle$ of $\mathcal{H}_{\text{MF}}$ has the special property $\mathcal{L}|g\rangle = 0$. And according to the above relations, this implies that $\langle g|S_1^+ S_2^-|g\rangle = 0$, which is equivalent with $m = 0$.

- For $S = 1$ and higher, the ground state does not obey the property $\mathcal{L}|g\rangle = 0$ and m is therefore finite. The numerical solution for $S = 1$ gives $h_{\text{loc}} = 0.83643$ and $m = 0.0011412$, which is a very small number.

- In the large-S limit, the parameter m must eventually vanish (consistent with the numerical results from the decoupled, large-S spin-wave Hamiltonian). The reason behind this is that as we increase S, the ground state $|g\rangle$ comes closer and closer to the classical vacuum $|0\rangle$ (with spins fully polarized along their local $\mathbf{w}$-axes), which has the property $\mathcal{L}|0\rangle = 0$ (because $|0\rangle$ is an eigenstate of each S_ν^w individually). In fact, this relation remains true when we include the leading effect of semiclassical corrections coming from $\mathcal{V}$. At this leading level, the ground state wavefunction is given by [4]

$$|g_1\rangle = |0\rangle + \mathcal{R}\mathcal{V}|0\rangle\,, \tag{42}$$

where $\mathcal{R} = \frac{1-|0\rangle\langle 0|}{E_0 - \mathcal{H}_0}$ is the usual resolvent operator. To show that $\mathcal{L}|g_1\rangle = 0$ we use the fact that $\mathcal{L}$ commutes with $\mathcal{H}_0$ (and therefore with $\mathcal{R}$ as well) and furthermore $\mathcal{L}|0\rangle = 0$. These properties give:

$$\mathcal{L}|g_1\rangle = \mathcal{L}|0\rangle + \mathcal{L}\mathcal{R}\mathcal{V}|0\rangle = \mathcal{R}\mathcal{L}\mathcal{V}|0\rangle = \mathcal{R}[\mathcal{L}, \mathcal{V}]|0\rangle\,. \tag{43}$$

We further have:

$$\begin{aligned} \mathcal{V} &= \frac{1}{4}\left(S_1^+ S_2^+ - S_2^+ S_3^+ + S_3^+ S_4^+ - S_4^+ S_5^+ + S_5^+ S_6^+ + \gamma S_6^+ S_1^+ + h.c.\right) \\ &+ \frac{1}{4}\left(S_1^+ S_2^- + S_2^+ S_3^- + S_3^+ S_4^- + S_4^+ S_5^- + S_5^+ S_6^- - \gamma S_6^+ S_1^- + h.c.\right) \equiv \mathcal{V}_1 + \mathcal{V}_2\,. \end{aligned} \tag{44}$$

Using the standard spin commutation relations we find

$$[\mathcal{L},\mathcal{V}_1]=0,\ \text{ and }\ [\mathcal{L},\mathcal{V}]=[\mathcal{L},\mathcal{V}_2]=\frac{1}{2}\left(S_1^+S_2^-+S_2^+S_3^-+\cdots-\gamma S_6^+S_1^-\right)-h.c.\,, \tag{45}$$

from which it follows that $[\mathcal{L},\mathcal{V}]|0\rangle=0$ and therefore $\mathcal{L}|g_1\rangle=0$.

At higher orders $n>1$, the ground state $|g_n\rangle$ does not satisfy this property (i.e. $\mathcal{L}|g_n\rangle\neq 0$), and a finite m is therefore expected (as found explicitly for $S=1$ above, by the exact treatment of the equivalent spin Hamiltonian $\mathcal{H}_{\text{MF}}$). Nevertheless, the important point is that m vanishes asymptotically for large S, and it is generally a very small number otherwise ($m=0.0011412$ at $S=1$).

Two-fold degeneracy structure of the spin-wave spectrum

Fig. 5 of the main text shows that the six spin-wave energies organize into three degenerate pairs. The symmetry origin of this degeneracy can be seen by considering the effect of the operation $\mathbf{\Sigma}_1$ discussed above. We have:

$$\mathbf{B}=\mathbf{S}^{-1}\cdot\mathbf{C}=\mathbf{g}\cdot\mathbf{S}^{+}\mathbf{g}\cdot\mathbf{C}\Rightarrow\mathbf{g}\cdot\mathbf{B}=\mathbf{S}^{+}\mathbf{g}\cdot\mathbf{C} \tag{46}$$

Let us take the first row of this matrix equation:

$$b_1=\mathbf{X}_1^{*}\cdot\left(c_1,c_2,c_3,c_4,c_5,c_6,-c_1^{+},-c_2^{+},-c_3^{+},-c_4^{+},-c_5^{+},-c_6^{+}\right) \tag{47}$$

Suppose further that $\mathbf{X}_1=(a_1,a_2,a_3,a_4,a_5,a_6,a_1',a_2',a_3',a_4',a_5',a_6')$. Now, if b_1 describes an eigenmode, then $\mathbf{\Sigma}_1\cdot b_1$ is also an eigenmode with the same energy:

$$\begin{aligned}&\mathbf{\Sigma}_1\cdot b_1=\\&=(a_1,a_2,a_3,a_4,a_5,a_6,a_1',a_2',a_3',a_4',a_5',a_6')^{*}\cdot\left(-ic_4,-ic_5,-ic_6,i\gamma c_1,i\gamma c_2,i\gamma c_3,-ic_4^{+},-ic_5^{+},-ic_6^{+},i\gamma c_1^{+},i\gamma c_2^{+},i\gamma c_3^{+}\right)\\&=-i(-a_4,-a_5,-a_6,a_1,a_2,a_3,a_4',a_5',a_6',-a_1',-a_2',-a_3')^{*}\cdot\left(\gamma c_1,\gamma c_2,\gamma c_3,c_4,c_5,c_6,-\gamma c_1^{+},-\gamma c_2^{+},-\gamma c_3^{+},-c_4^{+},-c_5^{+},-c_6^{+}\right)\\&\equiv -i\mathbf{X}_1'^{\,*}\cdot\left(c_1,c_2,c_3,c_4,c_5,c_6,-c_1^{+},-c_2^{+},-c_3^{+},-c_4^{+},-c_5^{+},-c_6^{+}\right)\end{aligned} \tag{48}$$

This means that the eigenvectors corresponding to the positive (or the negative) eigenvalues of $\mathbf{g}\cdot\mathbf{M}$ come in pairs:

$$\begin{aligned}\mathbf{X}_1&=(a_1,a_2,a_3,a_4,a_5,a_6,a_1',a_2',a_3',a_4',a_5',a_6')\\\mathbf{X}_1'&=(-\gamma a_4,-\gamma a_5,-\gamma a_6,a_1,a_2,a_3,\gamma a_4',\gamma a_5',\gamma a_6',-a_1',-a_2',-a_3')\,.\end{aligned} \tag{49}$$

If these modes are linearly independent they belong to a 2-dimensional irreducible representation of the symmetry group generated by $\mathbf{\Sigma}_1$-$\mathbf{\Sigma}_5$. The results show that this is the case for the whole spectrum of the spin-wave Hamiltonian.

Supplementary Note 2. Semiclassical expansion around the states of the staggered dimer pattern

For the numerical data shown in Fig. 7 **d** of the main text we have also performed a non-linear semiclassical expansion around the classical state associated with the staggered pattern of Fig. 7 **c** of the main text. In this pattern, the dimers occupy the horizontal, 'zz' bonds, while the empty bonds form infinite strings. Similarly to the above, the strings decouple from each other and it suffices to consider one string only. To this end we use the labeling scheme of Supplementary Figure 2 and the following local frames:

$$\begin{aligned}&(\mathbf{u}_1,\mathbf{v}_1,\mathbf{w}_1)=(\mathbf{x},\eta_1\mathbf{y},\eta_1\mathbf{z}),\ \ (\mathbf{u}_2,\mathbf{v}_2,\mathbf{w}_2)=(\mathbf{x},\eta_2\kappa\mathbf{y},\eta_2\kappa\mathbf{z}),\\&(\mathbf{u}_3,\mathbf{v}_3,\mathbf{w}_3)=(\eta_2\eta_3\kappa\mathbf{x},\eta_2\kappa\mathbf{y},\eta_3\mathbf{z}),\ \ (\mathbf{u}_4,\mathbf{v}_4,\mathbf{w}_4)=(\eta_2\eta_3\kappa\mathbf{x},\eta_2\eta_3\eta_4\mathbf{y},\eta_4\kappa\mathbf{z}),\end{aligned} \tag{50}$$

and so on. With this choice of local axes we move all the dependence on η's on the last bond at infinity. And since the string is infinite, the energy contribution from that last bond does not matter, and therefore the spin wave expansion

does not depend on the configuration of η's altogether. The Hamiltonian for the terms along the string becomes

$$\mathcal{H} = K(S_1^u S_2^u + S_2^v S_3^v + S_3^u S_4^u + \cdots) - |K|(S_1^w S_{1'}^w + S_2^w S_{2'}^w + \cdots) \tag{51}$$

The Hamiltonian along the string describe a system with a unit cell of two sites, and we can relabel the sites as follows:

$$1 \to (R=0, \nu=1),\ \ 2 \to (R=0, \nu=2),\ \ 3 \to (R=1, \nu=1),\ \ 4 \to (R=1, \nu=2)\,, \tag{52}$$

and so on. Keeping only the terms pertaining to the given string and going to momentum space (along the string) gives, in matrix notation:

$$\mathcal{H}/|K| = f_0 + \frac{1}{2}\sum_k \mathbf{C}_k^+ \cdot \mathbf{M}_k \cdot \mathbf{C}_k \tag{53}$$

where

$$\mathbf{C}_k^+ = \left(c_{k,1}^+, c_{k,2}^+, c_{-k,1}, c_{-k,2}\right), \quad \mathbf{M}_k = \left(\begin{array}{cc|cc} d & \chi_{12}(k) & d' & \rho_{12}(k) \\ \chi_{12}(-k) & d & \rho_{12}(-k) & d' \\ \hline d' & \rho_{12}(k) & d & \chi_{12}(k) \\ \rho_{12}(-k) & d' & \chi_{12}(-k) & d \end{array}\right), \tag{54}$$

and

$$\begin{gathered} f_0 = \left[p^2 - S^2 - \kappa(\tau_{12}+\tau'_{12}) - d\right] N_s/2, \quad d = (S-p) - 4\kappa(f_{12}-f'_{12}), \quad d' = -2\kappa(f_{12}+f'_{12}), \\ \rho_{12}(k) = -\kappa(g_{12} + g'_{12} e^{ik}), \quad \chi_{12}(k) = -\kappa(g_{12} - g'_{12} e^{ik})\,, \end{gathered} \tag{55}$$

where the constants f_{12}, f'_{12}, g_{12}, g'_{12}, τ_{12} and τ'_{12} are defined again as in (8) and (10) above, and N_s is the number of sites along the string. Here, the matrix $\mathbf{S}_k$ must satisfy:

$$\mathbf{S}_k^+ \cdot \mathbf{g} \cdot \mathbf{S}_k = \mathbf{g}, \quad \mathbf{S}_k = \begin{pmatrix} \mathbf{A}_k & \mathbf{B}_{-k}^* \\ \mathbf{B}_k & \mathbf{A}_{-k}^* \end{pmatrix} = \mathbf{S}_{-k}^*\,. \tag{56}$$

Note that the second relation replaces the relation $\mathbf{L}\cdot\mathbf{S}\cdot\mathbf{L} = \mathbf{S}^*$ that we had in (23) above.

Symmetry constraints

- *Symmetry* $\mathbf{\Sigma}'_1$. This is a translation by one lattice spacing, followed by a $\frac{\pi}{2}$-rotation around the local $\mathbf{w}$-axes:

  $$(S_{R,1}^u, S_{R,1}^v, S_{R,1}^w) \to (S_{R,2}^v, -S_{R,2}^u, S_{R,2}^w), \quad (S_{R,2}^u, S_{R,2}^v, S_{R,2}^w) \to (S_{R+1,1}^v, -S_{R+1,1}^u, S_{R+1,1}^w)\,,$$

  which is equivalent with

  $$\boxed{c_{R,1} \to -i c_{R,2}, \quad c_{R,2} \to -i c_{R+1,1}}\,. \tag{57}$$

- *Symmetry* $\mathbf{\Sigma}'_2$. This is a reflection though the bond (2,2') (see Supplementary Figure 2), followed by a $\frac{\pi}{2}$-rotation around the local $\mathbf{w}$-axes:

  $$(S_{R,1}^u, S_{R,1}^v, S_{R,1}^w) \to (S_{R+1,1}^v, -S_{R+1,1}^u, S_{R+1,1}^w), \quad (S_{R,2}^u, S_{R,2}^v, S_{R,2}^w) \to (S_{R,2}^v, -S_{R,2}^u, S_{R,2}^w)\,, \tag{58}$$

  which is equivalent with

  $$\boxed{c_{R,1} \to -i c_{R+1,1}, \quad c_{R,2} \to -i c_{R,2}}\,. \tag{59}$$

- *Symmetry* $\mathbf{\Sigma}'_3$. This is an inversion through the middle of the bond (1,2) (see Supplementary Figure 2), which maps $\boxed{c_{R,1} \to c_{R,2}}$.

  Combining the symmetries $\mathbf{\Sigma}'_1$-$\mathbf{\Sigma}'_3$ gives:

$$\boxed{\langle c_{R,1} c_{R,2}\rangle = -\langle c_{R,2} c_{R+1,1}\rangle = \cdots}, \quad \boxed{\langle c_{R,\nu} c_{R,\nu}\rangle = 0}, \quad \boxed{\langle c_{R,1} c_{R,2}^+\rangle = \langle c_{R,2} c_{R+1,1}^+\rangle = \cdots}\,. \tag{60}$$

Supplementary References

[1] T. Holstein and H. Primakoff, Phys. Rev. **58**, 1098 (1940).

[2] G. Baskaran, D. Sen, and R. Shankar, Phys. Rev. B **78**, 115116 (2008).

[3] J.-P. Blaizot and G. Ripka, *Quantum Theory of Finite Systems* (Cambridge, MA, 1986) Chap. 3.

[4] I. Lindgren, J. Phys. B: Atom. and Molec. Phys. **7**, 2441 (1974).
